# Comparative analysis of *Zanthoxylum armatum* essential oils from four cultivars: Chemical compositions, antioxidant activities, and antibacterial activities

**DOI:** 10.1016/j.fochx.2025.103005

**Published:** 2025-09-06

**Authors:** Shao-jun Fan, Wen-zhang Qian, Yi-xiao Xiao, Yun-yi Hu, Meng-lin Jiang, Ji-cheng Chen, Dan-ju Zhang, Shun Gao

**Affiliations:** a Department of Forestry, Faculty of Forestry, Sichuan Agricultural University, Chengdu, 611130, China; b School of Food Science and Technology, Jiangnan University, 214122 Wuxi, Jiangsu, China; c College of Food Science, Fujian Agriculture and Forest University, Fuzhou 350002, China; d Forest Ecology and Conservation in the Upper Reaches of the Yangtze River Key Laboratory of Sichuan Province & Sichuan Mt. Emei Forest Ecosystem National Observation and Research Station, Sichuan Agricultural University, Chengdu 611130, China

**Keywords:** *Zanthoxylum armatum*, Essential oil, Component identification, Antioxidant activity, Antibacterial activity, Network pharmacology, Molecular docking

## Abstract

Chemical compositions, antioxidant activities and antibacterial activities of essential oils (EOs) extracted from four *Zanthoxylum armatum* cultivars were comparatively analyzed. Results showed that EO yield of *Ziyang Qinghuajiao* (ZQ) (3.82 ± 0.16 %) fruits was significantly higher than those of *Tengjiao* (TJ), *Jiuyeqing* (JYQ), and *Yehuajiao* (YHJ). Approximately 90 volatile organic compounds (VOCs) were identified, with TJEOs and JYQEOs exhibiting similar chemical profiles that differed significantly from those of ZQEOs and YHJEOs. ZQEO demonstrated superior antioxidant activity and antidiabetic potential due to higher β-phellandrene and eucalyptol contents. TJEO and JYQEO exhibited stronger antibacterial activity against *Escherichia coli*, *Staphylococcus aureus*, and *Bacillus subtilis*, correlating with higher d-limonene levels. Network pharmacology and molecular docking suggested that d-limonene and β-phellandrene might exert antibacterial effects by inhibiting genetic material replication/expression and altering cell membrane permeability. These findings offered valuable insights for potential applications of these EOs in the food, agricultural, and pharmaceutical industries.

## Highlights

• • VOCs profiles and bioactivities of ZAEOs with marked cultivar-dependent variations.
• • ZQEO shows superior antioxidant and antidiabetic activities.
• • TJEO and JYQEO exhibit higher antibacterial activity linked to d-limonene levels.
• • Molecular docking revealed antimicrobial effects of d-limonene and β-phellandrene.
• • ZAEOs showed promising potential as natural antioxidants and antimicrobial agents.

## Graphical abstract

**Figure:**
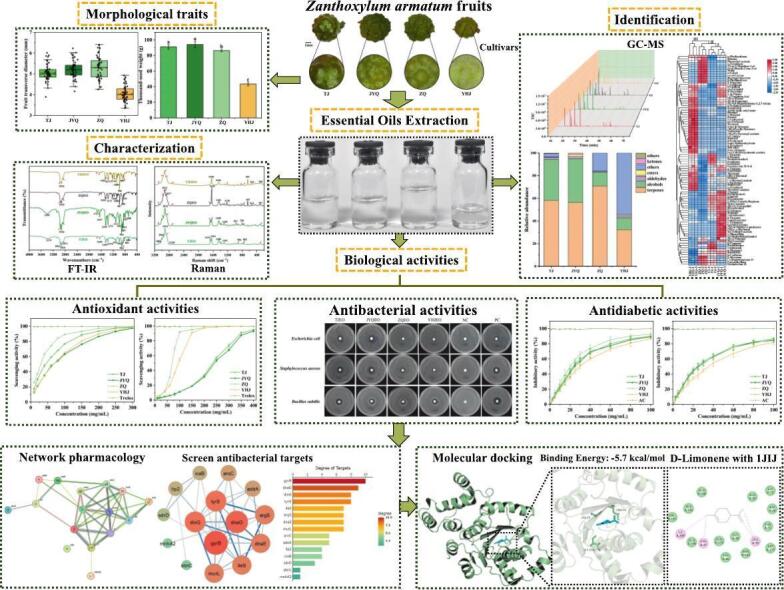

## 1 Introduction

Essential oils (EOs), complex mixtures of plant-derived volatile secondary metabolites including terpenoids, phenolics, alcohols, and derivatives (Falleh et al., 2020), are synthesized by plants for defense against pathogens, herbivores, and environmental stress, also aiding in allelopathy and communication (Raut & Karuppayil, 2014). Their chemical complexity, stemming from diverse biosynthetic pathways influenced by genetic, environmental, and developmental factors, confers a broad spectrum of functional properties such as antimicrobial, antioxidant, antifungal, and anti-inflammatory activities (Hou et al., 2022; Raut & Karuppayil, 2014). This makes EOs valuable resources in various industries. Recently, EOs have gained significant attention as natural alternatives to synthetic preservatives in food applications, inhibiting microbial growth and lipid oxidation (Zou et al., 2025). Furthermore, their use in postharvest treatments of fruits and vegetables extends shelf life, maintains quality, and reduces spoilage and waste. EOs are also increasingly utilized in functional foods, contributing health benefits aligned with consumer demand for natural nutraceuticals (Asbahani et al., 2015; Snyder et al., 2024). However, inherent compositional variability due to plant species, variety, and growth conditions limits EO application. Therefore, extracting EOs from diverse plant species, analyzing their components, and evaluating biological activities are crucial for broader food industry utilization.

The content, chemical composition, and bioactivity of EOs are dynamically influenced by a combination of intrinsic and extrinsic factors, which collectively shape their functional properties and industrial potential (Karalija et al., 2022; Meenu et al., 2023). Intrinsic factors, such as genetic background, plant species, cultivar, tissue type, etc., play a fundamental role in determining the biosynthetic pathways and metabolic profiles of EO. Genetic polymorphism and species-specific biosynthetic enzymes dictate the types and ratios of volatile compounds produced, leading to significant variability even within taxa (Huang et al., 2024), as evidenced by distinct chemotypes within a single species. Furthermore, developmental stages, such as flowering or fruiting, alter EO composition quantitatively and qualitatively, since secondary metabolite production is synchronized with physiological processes (Akachoud et al., 2024). Extrinsic factors, encompassing environmental conditions (e.g., temperature, light, soil, water, altitude), agricultural practices (e.g., fertilization, irrigation, harvest timing), and post-harvest processing (e.g., extraction methods), further modulate EO characteristics by influencing enzymatic activities and metabolic fluxes (Asbahani et al., 2015; Karalija et al., 2022). The complex interplay of these factors creates substantial EO variability, posing challenges for standardization. While this diversity offers opportunities for tailored applications across various sectors, it necessitates a deeper understanding of the key drivers of EO composition to ensure consistent quality and efficacy.

*Zanthoxylum armatum* DC*.* (*Z. armatum*), commonly known as “Qinghuajiao” in China, is a deciduous or evergreen thorny shrub or small tree, and a member of the Rutaceae family (Cheng et al., 2021). This plant is widely cultivated across Southwest China, India, Pakistan, Nepal, and other regions, where its green fruits have been harvested and utilized as a signature spice in Sichuan cuisine for over 2000 years (Liu et al., 2020). Beyond its culinary use, *Z. armatum* is valued for its EO content, characterized by high levels of limonene and linalool (Cheng et al., 2021), which impart distinctive flavor and aroma. Recent studies confirm the EO extracts exhibit significant biological activities, including antibacterial, antioxidant, and hypoglycemic effects (Wang et al., 2022), suggesting potential for pharmaceutical and cosmetic applications. However, significant variations in EO content and composition exist among *Z. armatum* populations across different geographical regions in China (Qian et al., 2024; Shao et al., 2023). The diverse chemical composition and bioactive properties of EO derived from *Z. armatum* fruits remain inadequately characterized and warrant further investigation. Herein, this study systematically investigated the volatile component profiles of EO from four *Z. armatum* cultivars (*Tengjiao*, *Jiuyeqing*, *Ziyang Qinghuajiao*, and *Yehuajiao*) using GC–MS analysis. Through integrated evaluation of antioxidant, antidiabetic, and antibacterial activities combined with OPLS-DA and Mantel test, the cultivar-specific correlations between phytochemicals and bioactivities also elucidated. The combination of network pharmacology and molecular docking further revealed the antibacterial mechanisms of key components. These findings provide scientific evidence for the rational utilization of *Z. armatum* EO in functional food and pharmaceutical development.

## 2 Materials and methods

### 2.1 Chemicals

2,2′-Azinobis (3-ethylbenzothiazoline-6-sulfonic acid) (ABTS) and 1,1-diphenyl-2-picrylhydrazyl (DPPH) were purchased from BASF BioScience Co., Ltd. (Hefei, China). Cyclohexanone (>99.9 %) and potassium persulfate were from Aladdin Biochemical Technology Co., Ltd. (Shanghai, China). n-Hexane, dimethyl sulfoxide (DMSO), anhydrous sodium sulfate, absolute ethanol, and soluble starch were from Chengdu Kelong Chemical Co., Ltd. (Chengdu, China). DNS chromogenic reagent, *p*-nitrophenyl-α-D-glucopyranoside (PNPG), and cefuroxime sodium were from Macklin Biochemical Co., Ltd. (Shanghai, China). C7-C30 n-alkanes, α-glucosidase, α-amylase, acarbose, Nutrient Agar, and Nutrient Broth were obtained from Sigma-Aldrich (St. Louis, MO, USA). Bacterial strains *Staphylococcus aureus* ATCC 6538, *Escherichia coli* ATCC 25922, and *Bacillus subtilis* ATCC 6633 were procured from the Shanghai Microbiological Culture Collection (SHMCC) Co., Ltd. (Shanghai, China). All chemicals were of analytical or chromatographic (GC) grade.

### 2.2 Plant materials and EO extraction

Fresh fruits from four *Z. armatum* cultivars, including *Tengjiao* (TJ), *Jiuyeqing* (JYQ), *Ziyang Qinghuajiao* (ZQ), and *Yehuajiao* (YHJ), were harvested in August 2024 in Yongan Town, Shuangliu District, Chengdu, China (30°22′10.09″N, 104°00′22.69″E, elevation 411–425 m). Detailed information on their differences is provided in Table S1 and Fig. S1. Fruits were collected, transported under cold chain conditions at 4 °C to the laboratory, and then manually separated, homogenized, and pooled for use. EO were extracted using microwave-assisted hydrodistillation, a method selected for its superior extraction efficiency, reduced thermal degradation of thermolabile compounds, and lower energy consumption compared to conventional hydrodistillation (Benmoussa et al., 2023; Singh Chouhan et al., 2019). Fruits (50 g per replicate, *n* ≥ 3) were immersed in 250 mL of deionized water for 3 h in a 500 mL round-bottom flask. The mixture was then subjected to a modified Clevenger apparatus operating at a microwave power of 640 W for 15 min, while maintaining the condenser temperature at 4 °C. The collected EO was dehydrated with anhydrous sodium sulfate, sealed, and stored at −18 °C until analysis. The EO yield (%, *w*/w) was calculated using the equation: EO yield (%) = EO mass (g) / fresh fruit mass (g) × 100. The resulting EO were designated as TJEO, JYQEO, ZQEO, and YHJEO.

### 2.3 Gas chromatography-mass spectrometry (GC–MS) analysis

*Z. armatum* essential oil (ZAEO) sample (4 μL) was transferred to a 2 mL GC vial, followed by the addition of 1 μL of cyclohexanone (internal standard, 0.948 g/mL), and then diluted to 2 mL with hexane to ensure homogeneity. Volatile compounds were analyzed using an Agilent 6890 A gas chromatograph coupled to an Agilent 5973C VL mass selective detector. Separation was performed on a non-polar HP-5 capillary column (60 m × 0.25 mm × 0.25 μm, Agilent Technologies, USA). The temperature program was: initial temperature 50 °C, increased at 2 °C per min to 150 °C (held for 2 min), then ramped at 6 °C per min to 250 °C (held for 8 min). Additional GC–MS parameters included: injector temperature 250 °C, carrier gas (helium) flow rate 0.99 mL/min, splitless injection mode with a splitless time of 0.8 min, interface temperature 250 °C, electron ionization energy 70 eV, ion source temperature 240 °C, and mass scan range 33–600 *m*/*z*. Compounds were identified by matching retention indices (RI) against a reference database and mass spectra against the NIST 2020 mass spectral library. In modern research, GC–MS has become a valid tool for RI determination due to its combined qualitative and quantitative capabilities, particularly for complex matrices like essential oils, with subsequent comparison to values reported in the literature (Bizzo et al., 2023; Xia et al., 2025). The RI of each component was calculated using the retention indices of n-alkanes (C7–C30) and the following Eq. (1):

(1) $$\mathrm{RI}=100\;n+\frac{100\;\left[t_{R}\left(x\right)-t_{R}\left(n\right)\right]}{\left[t_{R}\left(n+1\right)-t_{R}\left(n\right)\right]}$$

where: t_R_: retention time, x: the compound to be analyzed, n, n + 1: the number of carbon atoms of the two n-alkanes before and after the peak of the analyte to be analyzed, i.e., t_R_ (n) < t_R_ (x) < t_R_ (n + 1). The t_R_ values of these n-alkanes were determined separately under identical chromatographic conditions to ensure a comparable basis for RI calculation.

Quantification was performed using the internal standard method based on established protocols (Liu et al., 2023; Wang et al., 2025). The content of component *C*_i_ in the sample was calculated according to Eq. (2):

(2) $$C_{i}=\frac{A_{i}}{A_{s}\times m}\times W_{s}$$

where *C*_i_ is the relative concentration of the target volatile compounds (mg/g), representing milligrams of analyte per gram of sample; *A*_i_ is the peak area corresponding to the target volatile compounds; *A*_s_ is the peak area corresponding to the internal standard; *m* is the sample mass (g); *W*_s_ is the mass of the internal standard added (mg).

### 2.4 Multivariate analysis of volatile compounds

A flavoromics-based multivariate approach was used to identify key differential components in ZAEOs. Principal component analysis (PCA) was applied to analyze and visualize compositional variations among the four ZAEOs, aiming to assess the effect of extraction methods on ZAEO compositional profiles. Upset plots and Venn diagrams were generated to visually compare shared and unique volatile compounds across ZAEOs derived from different extraction methods.

Volcano plots were constructed using fold change (FC) and Welch's *t*-test (*p* < 0.05) thresholds, where compounds meeting *p* < 0.05 with log₂FC > 1 were defined as significantly upregulated, while those with *p* < 0.05 and log₂FC < −1 were classified as downregulated (Zhao et al., 2025). Orthogonal partial least squares-discriminant analysis (OPLS-DA) was further conducted, with variable importance in projection (VIP) scores >1.0 serving as the criterion for identifying discriminative compounds. Components fulfilling both VIP >1.0 and volcano plot significance thresholds were provisionally identified as key differential volatile compounds.

### 2.5 Fourier transform infrared spectroscopy (FT-IR) analysis

FT-IR spectra were recorded using the liquid film method with a Nicolet iS5 FT-IR spectrometer (Thermo Fisher Scientific, Waltham, MA, USA). Prior to analysis, blank potassium bromide (KBr) pellets were prepared and stored in a desiccator. For sample preparation, the EO were uniformly coated onto the KBr pellets. Spectral data were collected o*v*er a wavenumber range of 400–4000 cm^−1^ with a resolution of 4 cm^−1^, with 32 scans accumulated per measurement to optimize signal-to-noise ratio.

### 2.6 Raman spectroscopy analysis

The Raman spectra were acquired using a HORIBA HR Evolution Raman spectrometer (HORIBA Scientific, Kyoto, Japan). The measurement conditions were configured as follows: excitation wavelength of 785 nm, laser power of 1 mW, integration time of 60 s, and spectral range spanning from 200 cm^−1^ to 3200 cm^−1^. All measurements were conducted under ambient laboratory conditions with consistent instrument parameters to ensure reproducibility.

### 2.7 Antioxidant activity assay

#### 2.7.1 ABTS radical scavenging assay

The ABTS radical scavenging capacity was evaluated following a modified protocol adapted from Rumpf et al. (2024). Briefly, 7.0 mM ABTS solution and 2.45 mM potassium persulfate solution were mixed at a 1:1 (*v*/v) ratio and incubated in the dark at room temperature for 16 h to generate ABTS radicals. For the assay, 200 μL of EO (diluted in anhydrous ethanol) was combined with 3 mL of the activated ABTS solution. After 6 min of dark incubation at ambient temperature, absorbance was measured at 734 nm using a UV–Vis spectrophotometer. The scavenging rate (%) was calculated as follows: ABTS scavenging rate = (1 – *A*_sample_ / *A*_blank_) × 100, where *A*_blank_ represents the absorbance of anhydrous ethanol (negative control) and *A*_sample_ denotes the absorbance of the test solution. Trolox was used as the positive control.

#### 2.7.2 DPPH radical scavenging assay

DPPH radical scavenging activity was determined using a modified method from Ge et al. (2022). A 100 μL aliquot of EO was diluted with 900 μL anhydrous ethanol, followed by the addition of 2 mL of 0.06 mM DPPH ethanolic solution. The mixture was vortexed, incubated in the dark at 25 °C for 2 h, and centrifuged (5000 ×*g*, 10 min) to remove precipitated lipids. Absorbance was recorded at 517 nm. The scavenging rate (%) was calculated as follows: DPPH scavenging rate = (1 – *A*_sample_ / *A*_blank_) × 100. Anhydrous ethanol served as the blank (*A*_blank_), and Trolox was used as the positive control.

### 2.8 In vitro hypoglycemic activity assay

#### 2.8.1 Α-Glucosidase inhibitory activity assay

The α-glucosidase inhibitory activity was assessed using an in vitro enzymatic assay. EO samples and the positive control (acarbose) were dissolved in DMSO to obtain stock solutions of appropriate concentrations. The inhibitory assay was performed according to a protocol adapted from Zhang et al. (2017) with modifications. Specifically, 100 μL of sample solution (1–40 mg/mL) was combined with 20 μL of 1 U/mL α-glucosidase (prepared in 0.1 M phosphate buffer, pH 6.8) in a 96-well plate. Following a 20-min pre-incubation at 37 °C, 100 μL of 4 mM *p*-nitrophenyl-α-D-glucopyranoside (PNPG) substrate solution was added to initiate the reaction. After 10 min of incubation at 37 °C, the enzymatic reaction was terminated by adding 100 μL of 0.1 M sodium carbonate (Na_2_CO_3_). Absorbance was measured at 405 nm using a microplate reader. The inhibition rate was calculated using the formula: Inhibition rate (%) = [(*A*c − *A*_0_) − (*A*_1_ − *A*)] / (*A*c − *A*_0_) × 100, where *A*₁ denotes the absorbance of the sample, *A*c is the absorbance of the control, *A*_0_ is the absorbance of the blank sample, and *A* represents the background absorbance.

#### 2.8.2 Α-Amylase inhibition acti*v*ity assay

The α-amylase inhibitory activity was determined following the method of Jaradat et al. (2020) with optimization. Briefly, 150 μL of sample solution (1–40 mg/mL) was mixed with 50 μL of 2 U/mL α-amylase (in 0.02 M phosphate buffer, pH 6.9) and pre-incubated at 30 °C for 30 min. Subsequently, 100 μL of 1 % (*w*/*v*) soluble starch solution was added, and the reaction mixture was incubated at 37 °C for 3 min. To terminate the reaction, 100 μL of 3,5-dinitrosalicylic acid (DNS) reagent was added, followed by dilution with 3.6 mL distilled water. The mixture was heated at 95 °C for 10 min in a water bath, cooled to ambient temperature, and absorbance was recorded at 540 nm. Acarbose was used as a reference inhibitor. The inhibition of EO in the α-amylase reaction was calculated as follows: Inhibition rate (%) = [(*A*c − *A*_0_) - (*A*_1_ − *A*)] / (*A*c − *A*_0_) × 100 %, where *A*c represents the absorbance of the control, *A*_0_ represents the absorbance of the blank sample, *A*_1_ represents the absorbance of the sample, and *A* represents the background absorbance.

### 2.9 Antibacterial activity assay

The antibacterial activity of EO was evaluated against three microbial strains: *Staphylococcus aureus* (ATCC 6538), *Escherichia coli* (ATCC 25922), and *Bacillus subtilis* (ATCC 6633). A modified disc diffusion method (Li et al., 2022) was employed for initial screening. Briefly, 100 μL of bacterial suspension (approximately 10^6^–10^7^ CFU/mL) was uniformly spread on nutrient agar (NA) plates. Sterilized antimicrobial discs (6 mm diameter) were placed on the agar surface. Test groups received 10 μL of EO applied to filter discs, while control groups consisted of discs treated with dimethyl sulfoxide (DMSO) alone (negative control) and 0.25 mg/mL cefuroxime sodium (positive control). All plates were incubated inverted at 37 °C for 24 h, with triplicate experiments conducted. Inhibition zone diameters (DIZ) were measured using digital calipers.

The minimum inhibitory concentration (MIC) and minimum bactericidal concentration (MBC) were determined using the broth microdilution method (Wang, Zhou, et al., 2024; Xing et al., 2024). Briefly, 100 μL of sterile broth medium was dispensed into each well of a 96-well microtiter plate. Antimicrobial solutions were added to the first well of each row. Subsequently, two-fold serial dilutions were performed across the plate and mixed thoroughly (with the dilution series prepared in DMSO, with final concentrations ranging from 100 to 1.5625 mg/mL). Next, 100 μL of bacterial suspension was added to each well, achieving a final inoculum density of approximately 10^5^ CFU/mL. The microtiter plate was then incubated at 37 °C for 24 h. Following incubation, the optical density (OD_600_) of each well was measured at 600 nm using a microplate reader (SpectraMax M2e, Molecular Devices, Shanghai, China). Wells containing the bacterial suspension without antimicrobial agents served as the positive growth control. The MIC was defined as the lowest concentration of antimicrobial agents at which no visible bacterial growth was observed in the well (OD_600_ value change <0.05). For MBC determination, 100 μL aliquots from wells showing no visible growth (containing antimicrobial agent concentrations ≥ MIC) were aspirated, spread evenly onto NA plates, and incubated at 37 °C for 24 h. The MBC was recorded as the lowest antimicrobial concentration that resulted in no bacterial colony formation on the agar plate.

### 2.10 Network pharmacology analysis

Network pharmacology analysis was performed as follows. To predict compound-target interactions, SMILES notations of compounds were retrieved from PubChem (https://pubchem.ncbi.nlm.nih.gov/), and complete protein sequences of *Escherichia coli* and *Staphylococcus aureus* were obtained from UniProt (https://www.uniprot.org/). Potential antibacterial targets were predicted using the DeepPurpose algorithm (https://github.com/kexinhuang12345/DeepPurpose) with its pretrained model (model_DeepDTA_DAVIS), applying a high-affinity binding threshold of -log(Kd) ≥ 5.5 (Huang et al., 2021). Protein-protein interaction (PPI) networks for these targets were constructed using STRING (https://string-db.org/) under a minimum required interaction confidence score of 0.4 (medium confidence) (Szklarczyk et al., 2021). Resulting networks were imported into Cytoscape v3.10.1 and further filtered by applying a combined interaction score threshold ≥0.15 and removing disconnected (isolated) nodes. The NetworkAnalyzer tool was then used for interaction visualization and degree centrality calculation (Shannon et al., 2003). Functional enrichment analysis was subsequently performed in R using clusterProfiler, with statistical significance defined by an adjusted *p*-value (false discovery rate, FDR) < 0.05, leveraging gene sets from Gene Ontology (GO; https://geneontology.org/) and Kyoto Encyclopedia of Genes and Genomes (KEGG; https://www.genome.jp/kegg/).

### 2.11 Molecular docking

The three-dimensional (3D) protein structures were obtained from the Protein Data Bank (PDB, https://www.rcsb.org), and small molecule ligands were acquired from the PubChem database (https://pubchem.ncbi.nlm.nih.gov). Molecular docking was performed using AutoDock Vina software following these procedures: Both protein and ligand structures underwent essential preprocessing, with the entire protein surface designated as the potential active site. The docking grid was defined as a cubic box with 90 Å edge length and 0.375 Å spacing to encompass the complete protein structure. Conformational sampling was conducted through genetic algorithm optimization, with generated poses being ranked by their docking scores. The top-ranked conformation demonstrating the most favorable binding affinity was selected for subsequent interaction analysis. Post-docking visualization and binding mode interpretation were performed using PyMOL software (Version 3.0.3), enabling 3D characterization of molecular interactions between ligands and target proteins.

### 2.12 Statistical analysis

All measurements were performed in triplicate. Statistical significance was determined by one-way analysis of variance (ANOVA) followed by Duncan's multiple comparison test. A *p*-value <0.05 was considered statistically significant, while a *p*-value <0.01 was considered highly significant. Correlation matrices were visualized using heatmaps based on Pearson correlation coefficients. Hierarchical clustering analysis was also applied to the heatmaps to evaluate similarities and differences among EO components. Statistical analyses were conducted using Excel 2019 (Microsoft, USA) and SPSS 27.0 (IBM, USA), respectively. Graphs were created with Excel 2019 and Origin 2024b (OriginLab, USA). Orthogonal partial least squares-discriminant analysis (OPLS-DA) was performed using SIMCA-P 14.1 (Umetrics, Sweden).

## 3 Results and discussion

### 3.1 Morphological characteristics and EO yield

**Fig. 1:**
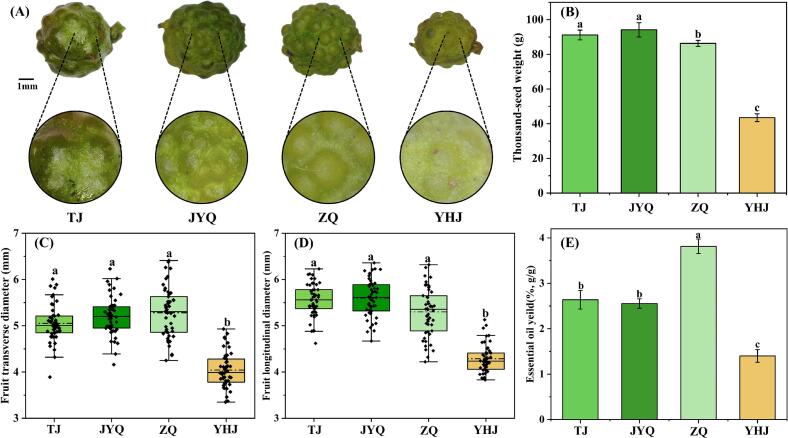
Morphological parameters and EO content of *Z. armatum* fruits from four cultivars. (A) The appearance of the fruits. (B) thousand-fruit weight. (C) fruit transverse diameter. (D) fruit longitudinal diameter. (E) EO yield. Different letters indicate significant differences (*p* < 0.05). TJ, *Tengjiao*. JYQ, *Jiuyeqing*. ZQ, *Ziyang Qinghuajiao*. YHJ, *Yehuajiao*.

This study revealed substantial phenotypic variations in fruits among four cultivars of *Z. armatum*, extending beyond basic fruit size and coloration to encompass differential accumulation of nutritional components and bioactive substances. Fig. 1A illustrated the distinct fruit morphologies observed among the four *Z. armatum* cultivars. Quantitative analysis demonstrated significant inter-cultivar differences in 1000-grain weight (Fig. 1B), transverse diameter (Fig. 1C), and longitudinal diameter (Fig. 1D). Notably, the 1000-grain weight varied considerably across cultivars, with TJ (91.18 ± 2.81 g) and JYQ (94.16 ± 4.12 g) showing comparable values, followed by ZQ (86.34 ± 1.73 g), while YHJ exhibited a markedly lower value (43.46 ± 2.28 g). Different patterns were also observed in dimensional measurements: transverse diameters ranged from 5.05 ± 0.40 mm (TJ) to 4.04 ± 0.37 mm (YHJ), and longitudinal diameters from 5.56 ± 0.37 mm (TJ) to 4.29 ± 0.30 mm (YHJ). These morphometric parameters demonstrated that YHJ fruits were significantly smaller than those of the other three cultivars, and this phenotypic trait may be associated with its unique genetic factors, phytochemical profile, and physiological characteristics (Tang, Wu, et al., 2023).

As shown in Fig. 1E, EO yield exhibited remarkable cultivar-dependent variations (1.40–3.82 %). ZQ produced the highest EO content (3.82 ± 0.16 %), followed by TJ (2.64 ± 0.21 %), JYQ (2.56 ± 0.10 %), and YHJ (1.40 ± 0.14 %). The superior EO yield in ZQ may be attributed to its well-developed secretory cavity (oil glands) with high secretory capacity, whereas the reduced productivity in YHJ could result from both smaller fruit size and underdeveloped secretory structures. This correlation between gland morphology and EO output aligns with previous observations in Rutaceae species (Wang, Ren, et al., 2024), indicating that oil glands density and developmental status represent one of the key factors affecting EO biosynthesis capacity. Zhao et al. (2023) reported significant differences in EO content (1.35 %–1.85 %) between two varieties of *Cinnamomum longepaniculatum*. In an investigation by Shao et al. (2023), a comparative assessment of terpenoid constituents was conducted across 48 huajiao samples collected from disparate geographic regions. The data revealed that volatile oil compositions exhibited variations influenced by a combination of factors including, but not limited to, cultivar lineage, ecological conditions, climatic conditions, and soil moisture levels. These findings suggested that cultivars exhibit considerable variations in EO yield, and the deliberate selection of appropriate genetic materials is paramount to ensure both maximum yield and high-quality EO production.

### 3.2 GC–MS analysis

**Fig. 2:**
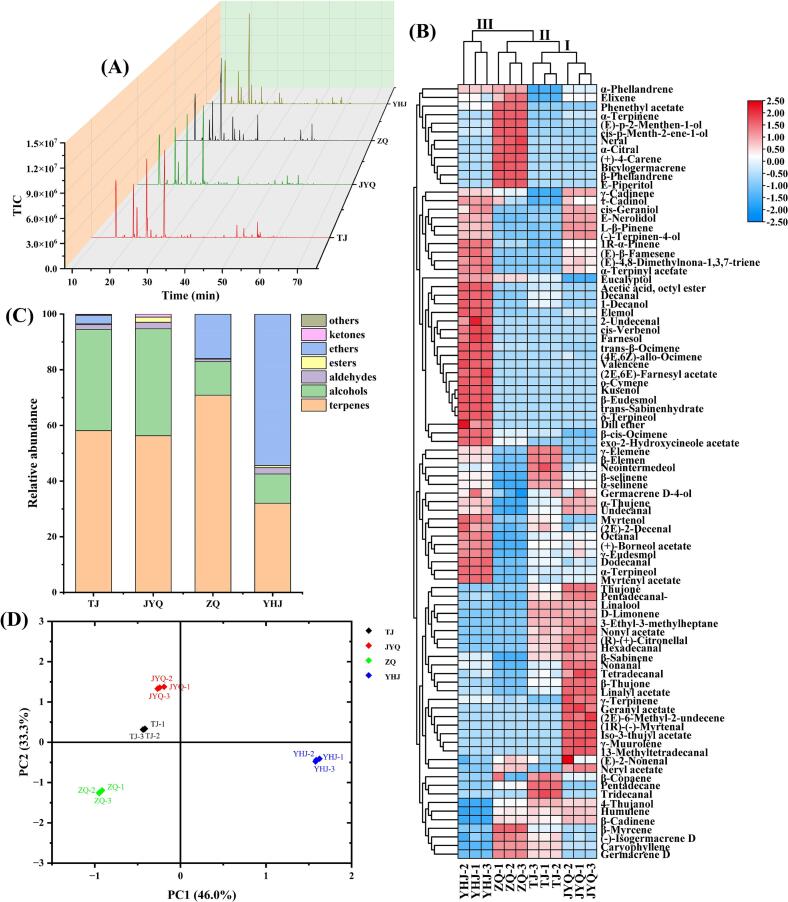
VOCs analysis of four ZAEOs. (A) Total ion chromatograms. (B) Heat map and cluster analysis of VOCs. (C) Relative percentage distribution of VOCs. (D) PCA score plot.

As illustrated in Fig. 2A and Table 1, 90 volatile organic compounds (VOCs), including terpenes (35), alcohols (21), aldehydes (17), esters (12), ethers (2), ketones (2), and one other compound (1) were identified. Significant differences in the VOC composition among four ZAEOs were observed. Both TJEO and JYQEO shared identical major components, including linalool, d-limonene, β-sabinene, and β-myrcene. In contrast, ZQEO primarily consisted of β-phellandrene, eucalyptol, β-myrcene, β-sabinene, and *E*-piperitol. YHJEO contained eucalyptol, β-sabinene, β-myrcene, β-*cis*-ocimene, and linalool as its predominant constituents. Significant compositional differences were observed across cultivars: ZQ (48 compounds) exhibited fewer VOCs compared to the other cultivars, with TJ (58 compounds) containing fewer compounds than JYQ (64 compounds). Notably, YHJEO had the highest number of volatile components (68 compounds) and eucalyptol content, while ZQEO displayed the highest β-phellandrene levels. TJEO and JYQEO were characterized by predominantly high contents of linalool and d-limonene. These variations may contribute to the distinct aromatic profiles and flavor release characteristics of four ZAEOs. Furthermore, our findings align with previous studies confirming linalool, d-limonene, β-sabinene, β-phellandrene, and eucalyptol as principal volatile constituents in ZAEO (Qian et al., 2024).

**Table 1:** The VOCs of four ZAEOs were identified by GC–MS.

No.	RI^a^	Compound	CAS	Formula	TJ (mg/g)^c^	JYQ (mg/g)^c^	ZQ (mg/g)^c^	YHJ (mg/g)^c^	Identification^b^
1	927	α-Thujene	2867–05-2	C_10_H_16_	2.57 ± 0.06	3.38 ± 0.08	1.65 ± 0.03	3.22 ± 0.05	RI, MS
2	934	1R-α-Pinene	7785-70-8	C_10_H_16_	7.74 ± 0.07	15.84 ± 0.12	10.59 ± 0.08	28.38 ± 0.21	RI, MS
3	974	β-Sabinene	3387-41-5	C_10_H_16_	148.76 ± 0.18	182.13 ± 1.27	65.71 ± 0.12	113.41 ± 0.41	RI, MS
4	978	L-β-Pinene	18,172–67-3	C_10_H_16_	8.40 ± 0.08	11.77 ± 0.09	8.48 ± 0.06	11.16 ± 0.15	RI, MS
5	991	β-Myrcene	123–35-3	C_10_H_16_	67.36 ± 0.11	56.94 ± 0.32	116.21 ± 0.34	50.47 ± 0.27	RI, MS
6	1003	Octanal	124–13-0	C_8_H_16_O	1.66 ± 0.04	1.77 ± 0.07	1.09 ± 0.04	2.42 ± 0.04	RI, MS
7	1006	α-Phellandrene	99–83-2	C_10_H_16_	1.25 ± 0.05	5.36 ± 0.13	12.47 ± 0.04	10.02 ± 0.07	MS
8	1018	α-Terpinene	99–86-5	C_10_H_16_	0.49 ± 0.02	0.98 ± 0.03	4.12 ± 0.04	0.75 ± 0.02	RI, MS
9	1026	o-Cymene	527–84-4	C_10_H_14_	–	–	–	0.53 ± 0.03	RI, MS
10	1031	d-Limonene	5989-27-5	C_10_H_16_	216.54 ± 0.64	210.90 ± 1.78	–	–	RI, MS
11	1033	β-Phellandrene	555–10-2	C_10_H_16_	–	–	338.55 ± 7.20	–	RI, MS
12	1034	Eucalyptol	470–82-6	C_10_H_18_O	29.49 ± 0.17	–	158.05 ± 9.53	533.14 ± 0.25	RI, MS
13	1037	trans-β-Ocimene	3779–61-1	C_10_H_16_	0.76 ± 0.02	0.56 ± 0.03	0.90 ± 0.09	4.86 ± 0.02	RI, MS
14	1048	β-cis-Ocimene	3338-55-4	C_10_H_16_	12.86 ± 0.05	7.42 ± 0.08	14.58 ± 0.07	39.13 ± 0.11	RI, MS
15	1057	3-Ethyl-3-methylheptane	17,302–01-1	C_10_H_22_	0.23 ± 0.00	0.27 ± 0.01	–	–	RI, MS
16	1060	γ-Terpinene	99–85-4	C_10_H_16_	0.99 ± 0.01	1.69 ± 0.07	1.13 ± 0.03	1.26 ± 0.02	RI, MS
17	1068	4-Thujanol	546–79-2	C_10_H_18_O	6.08 ± 0.02	4.64 ± 0.04	3.10 ± 0.12	–	RI, MS
18	1069	trans-Sabinenhydrate	17,699–16-0	C_10_H_18_O	–	–	–	8.67 ± 0.05	RI, MS
19	1090	(+)-4-Carene	29,050–33-7	C_10_H_16_	2.62 ± 0.02	2.30 ± 0.05	48.59 ± 0.23	3.25 ± 0.06	RI, MS
20	1100	Linalool	78–70-6	C_10_H_18_O	343.82 ± 1.49	354.74 ± 3.28	21.93 ± 0.18	35.28 ± 0.34	RI, MS
21	1105	Nonanal	124–19-6	C_9_H_18_O	3.54 ± 0.09	6.38 ± 0.05	2.16 ± 0.03	3.64 ± 0.11	RI, MS
22	1110	β-Thujone	471–15-8	C_10_H_16_O	1.71 ± 0.16	7.22 ± 0.02	–	0.47 ± 0.08	RI, MS
23	1117	(*E*)-4,8-Dimethylnona-1,3,7-triene	19,945–61-0	C_11_H_18_	–	0.58 ± 0.08	–	0.99 ± 0.10	RI, MS
24	1119	Thujone	546–80-5	C_10_H_16_O	1.20 ± 0.21	3.57 ± 0.08	–	–	RI, MS
25	1123	(E)-p-2-Menthen-1-ol	29,803–81-4	C_10_H_18_O	–	0.27 ± 0.07	19.99 ± 0.10	0.65 ± 0.05	RI, MS
26	1129	(4E,6Z)-allo-Ocimene	7216-56-0	C_10_H_16_	0.62 ± 0.07	0.66 ± 0.22	0.56 ± 0.02	3.40 ± 0.04	MS
27	1141	cis-p-Menth-2-ene-1-ol	29,803–82-5	C_10_H_18_O	–	–	12.21 ± 0.06	0.61 ± 0.02	RI, MS
28	1147	cis-Verbenol	1845-30-3	C_10_H_16_O	–	–	–	0.39 ± 0.05	RI, MS
29	1153	(*R*)-(+)-Citronellal	2385-77-5	C_10_H_18_O	0.98 ± 0.03	1.55 ± 0.07	–	–	RI, MS
30	1160	(E)-2-Nonenal	18,829–56-6	C_9_H_16_O	0.56 ± 0.07	0.73 ± 0.19	0.67 ± 0.03	0.48 ± 0.04	RI, MS
31	1166	(2E)-6-Methyl-2-undecene	74,630–61-8	C_12_H_24_	–	0.32 ± 0.04	–	–	RI, MS
32	1169	δ-Terpineol	7299-42-5	C_10_H_18_O	–	–	–	5.90 ± 0.17	RI, MS
33	1175	Nonyl acetate	143–13-5	C_11_H_22_O_2_	0.21 ± 0.03	0.30 ± 0.04	–	–	RI, MS
34	1180	(−)-Terpinen-4-ol	20,126–76-5	C_10_H_18_O	1.86 ± 0.03	2.98 ± 0.07	1.85 ± 0.04	2.74 ± 0.01	RI, MS
35	1189	Dill ether	74,410–10-9	C_10_H_16_O	–	–	–	0.51 ± 0.19	RI, MS
36	1193	α-Terpineol	98–55-5	C_10_H_18_O	3.05 ± 0.04	3.20 ± 0.02	0.55 ± 0.05	21.54 ± 0.09	RI, MS
37	1199	Myrtenol	515–00-4	C_10_H_16_O	0.92 ± 0.08	–	–	2.34 ± 0.19	RI, MS
38	1200	(1*R*)-(−)-Myrtenal	18,486–69-6	C_10_H_14_O	–	1.70 ± 0.17	–	–	RI, MS
39	1205	Decanal	112–31-2	C_10_H_20_O	5.03 ± 0.05	3.69 ± 0.07	3.30 ± 0.09	10.62 ± 0.07	RI, MS
40	1210	*E*-Piperitol	16,721–39-4	C_10_H_18_O	–	–	59.98 ± 0.35	–	RI, MS
41	1211	Acetic acid, octyl ester	112–14-1	C_10_H_20_O_2_	0.19 ± 0.01	–	–	1.85 ± 0.01	RI, MS
42	1221	Iso-3-thujyl acetate	62,181–90-2	C_12_H_20_O_2_	–	0.52 ± 0.02	–	–	RI, MS
43	1229	cis-Geraniol	106–25-2	C_10_H_18_O	–	0.56 ± 0.04	–	0.56 ± 0.16	RI, MS
44	1243	Neral	106–26-3	C_10_H_16_O	–	–	0.34 ± 0.04	–	RI, MS
45	1256	Linalyl acetate	115–95-7	C_12_H_20_O_2_	1.69 ± 0.29	15.20 ± 0.14	–	0.89 ± 0.03	RI, MS
46	1258	Phenethyl acetate	103–45-7	C_10_H_12_O_2_	–	–	0.82 ± 0.01	0.28 ± 0.02	RI, MS
47	1262	(2E)-2-Decenal	3913-81-3	C_10_H_18_O	0.88 ± 0.09	0.56 ± 0.10	0.39 ± 0.03	1.10 ± 0.13	RI, MS
48	1271	1-Decanol	112–30-1	C_10_H_22_O	0.37 ± 0.07	–	–	1.94 ± 0.07	RI, MS
49	1272	α-Citral	141–27-5	C_10_H_16_O	–	–	0.63 ± 0.04	–	RI, MS
50	1289	(+)-Borneol acetate	20,347–65-3	C_12_H_20_O_2_	0.28 ± 0.01	0.26 ± 0.06	–	0.48 ± 0.02	RI, MS
51	1307	Undecanal	112–44-7	C_11_H_22_O	1.05 ± 0.03	1.52 ± 0.08	0.40 ± 0.02	1.33 ± 0.13	RI, MS
52	1328	Myrtenyl acetate	1079-01-2	C_12_H_18_O_2_	0.39 ± 0.15	0.49 ± 0.02	–	1.75 ± 0.04	RI, MS
53	1342	γ-Elemene	29,873–99-2	C_15_H_24_	3.62 ± 0.03	–	–	1.69 ± 0.07	MS
54	1345	exo-2-Hydroxycineole acetate	57,709–95-2	C_12_H_20_O_3_	–	–	0.34 ± 0.01	1.26 ± 0.03	RI, MS
55	1352	α-Terpinyl acetate	80–26-2	C_12_H_20_O_2_	–	0.52 ± 0.09	–	0.93 ± 0.07	RI, MS
56	1364	Neryl acetate	141–12-8	C_12_H_20_O_2_	–	0.41 ± 0.04	0.30 ± 0.02	–	RI, MS
57	1364	2-Undecenal	2463-77-6	C_11_H_20_O	–	–	–	0.44 ± 0.14	RI, MS
58	1383	Geranyl acetate	105–87-3	C_12_H_20_O_2_	–	0.70 ± 0.12	–	–	RI, MS
59	1397	β-Elemene	515–13-9	C_15_H_24_	20.23 ± 0.04	3.45 ± 0.11	2.26 ± 0.05	10.21 ± 0.06	RI, MS
60	1409	Dodecanal	112–54-9	C_12_H_24_O	0.75 ± 0.01	0.55 ± 0.10	0.28 ± 0.01	1.62 ± 0.04	RI, MS
61	1428	Caryophyllene	87–44-5	C_15_H_24_	18.01 ± 0.08	9.15 ± 0.13	25.88 ± 0.13	4.61 ± 0.04	RI, MS
62	1438	β-Copaene	18,252–44-3	C_15_H_24_	2.66 ± 0.23	1.39 ± 0.03	1.40 ± 1.39	0.88 ± 0.01	MS
63	1441	Elixene	3242-08-8	C_15_H_24_	–	1.89 ± 0.04	4.87 ± 1.49	1.70 ± 0.59	RI, MS
64	1456	(−)-Isogermacrene D	317,819–80-0	C_15_H_24_	0.78 ± 0.04	0.53 ± 0.06	1.02 ± 0.04	0.40 ± 0.08	RI, MS
65	1462	(E)-β-Famesene	18,794–84-8	C_15_H_24_	–	0.45 ± 0.02	–	0.93 ± 0.06	RI, MS
66	1465	Humulene	6753-98-6	C_15_H_24_	5.48 ± 0.24	5.85 ± 0.09	5.12 ± 0.10	2.25 ± 0.08	RI, MS
67	1485	γ-Muurolene	30,021–74-0	C_15_H_24_	–	1.13 ± 0.05	–	–	RI, MS
68	1485	Valencene	4630-07-3	C_15_H_24_	–	–	–	0.67 ± 0.06	RI, MS
69	1491	Germacrene D	23,986–74-5	C_15_H_24_	21.22 ± 0.01	13.26 ± 0.12	27.61 ± 0.10	11.14 ± 0.07	RI, MS
70	1495	β-selinene	17,066–67-0	C_15_H_24_	4.16 ± 0.09	1.75 ± 0.03	0.41 ± 0.02	2.36 ± 0.08	RI, MS
71	1499	Pentadecane	629–62-9	C_15_H_32_	1.80 ± 0.01	–	0.56 ± 0.07	–	RI, MS
72	1504	α-selinene	473–13-2	C_15_H_24_	11.41 ± 0.13	4.92 ± 0.05	–	5.45 ± 0.10	RI, MS
73	1506	Bicylogermacrene	24,703–35-3	C_15_H_24_	–	–	4.44 ± 0.30	–	RI, MS
74	1512	Tridecanal	10,486–19-8	C_13_H_26_O	0.77 ± 0.07	–	–	–	RI, MS
75	1524	γ-Cadinene	39,029–41-9	C_15_H_24_	0.55 ± 0.01	2.06 ± 0.01	1.36 ± 0.02	1.76 ± 0.01	RI, MS
76	1533	β-Cadinene	523–47-7	C_15_H_24_	0.64 ± 0.04	0.82 ± 0.02	0.69 ± 0.02	–	RI, MS
77	1560	Elemol	639–99-6	C_15_H_26_O	0.77 ± 0.03	1.23 ± 0.02	0.66 ± 0.05	5.26 ± 0.06	RI, MS
78	1569	E-Nerolidol	40,716–66-3	C_15_H_26_O	0.47 ± 0.03	10.78 ± 0.10	–	9.12 ± 0.12	RI, MS
79	1578	13-Methyltetradecanal	75,853–51-9	C_15_H_30_O	–	0.50 ± 0.01	–	–	RI, MS
80	1587	Germacrene D-4-ol	198,991–79-6	C_15_H_26_O	0.86 ± 0.08	0.90 ± 0.11	0.64 ± 0.09	0.96 ± 0.10	RI, MS
81	1614	Tetradecanal	124–25-4	C_14_H_28_O	0.53 ± 0.14	2.01 ± 0.53	–	0.36 ± 0.15	RI, MS
82	1627	Neointermedeol	5945–72-2	C_15_H_26_O	0.84 ± 0.11	0.24 ± 0.10	–	0.29 ± 0.06	RI, MS
83	1636	γ-Eudesmol	1209-71-8	C_15_H_26_O	0.34 ± 0.05	0.42 ± 0.14	–	0.86 ± 0.04	RI, MS
84	1645	Kusenol	20,489–45-6	C_15_H_26_O	–	–	–	1.02 ± 0.04	RI, MS
85	1654	τ-Cadinol	5937-11-1	C_15_H_26_O	0.31 ± 0.03	1.19 ± 0.17	0.94 ± 0.40	1.68 ± 0.04	RI, MS
86	1667	β-Eudesmol	473–15-4	C_15_H_26_O	–	–	–	2.91 ± 0.11	RI, MS
87	1716	Pentadecanal-	2765-11-9	C_15_H_30_O	0.67 ± 0.03	1.19 ± 0.06	–	0.22 ± 0.05	RI, MS
88	1727	Farnesol	4602-84-0	C_15_H_26_O	–	–	–	0.41 ± 0.06	RI, MS
89	1818	Hexadecanal	629–80-1	C_16_H_32_O	0.41 ± 0.02	0.65 ± 0.07	–	–	RI, MS
90	1845	(2E,6E)-Farnesyl acetate	4128-17-0	C_17_H_28_O_2_	–	–	–	0.36 ± 0.04	RI, MS
Total volatile components					973.43 ± 1.70	980.90 ± 7.47	989.78 ± 2.81	982.15 ± 2.07	RI, MS

Abbreviations: MS – mass spectrum; RI – Programmed-temperature retention indices.

Relative percentage contents are shown as mean ± standard error. The number of experimental replicates was *n* = 3.

a Retention indices were determined by GC–MS on the stationary phase (HP-5).

b Identification: RI – compound confirmed by retention index; MS – compound identified using the NIST 2020 mass spectral database.

c “—” indicates a compound was not detected. Chemical component contents are expressed as mg/g, representing milligrams of target compounds per gram of sample.

#### 3.2.1 Analysis of cluster heat map and PCA

Fig. 2B illustrated the concentrations of VOCs in four ZAEOs through color gradients. The identified VOCs in four ZAEOs were categorized into three distinct clusters according to the dendrogram. TJEO and JYQEO clustered together as Group I, which exhibited lower contents of most individual compounds compared to other groups. ZQEO demonstrated closer chemical similarity to Group I, yet was ultimately classified into Group II. Notably, YHJEO exclusively formed Group III, indicating its markedly distinct VOC profile compared to the other two groups. Fig. 2C demonstrated that ZAEOs are primarily composed of terpenes, alcohols, aldehydes, esters, and ethers. Consistent with previous studies, terpenes and alcohols constitute critical aromatic components in ZAEO (Liu et al., 2020). In the present study, TJEO and JYQEO exhibited similar composition profiles across various components, while showing the most significant differences from YHJEO. This observation aligns well with the clustering analysis results presented earlier. As shown in Fig. 2D, PCA revealed effective discrimination of aroma components among the four ZAEOs. The cumulative contribution rate of the first two principal components reached 79.3 %, with PC1 and PC2 accounting for 46.0 % and 33.3 % of total variance, respectively. This clear separation pattern in PCA coordinates corresponded well with the hierarchical clustering results, suggesting significant inter-group variations in volatile composition that align with their botanical classifications.

#### 3.2.2 Analysis of upset plots, Venn diagrams, and volcano plots

**Fig. 3:**
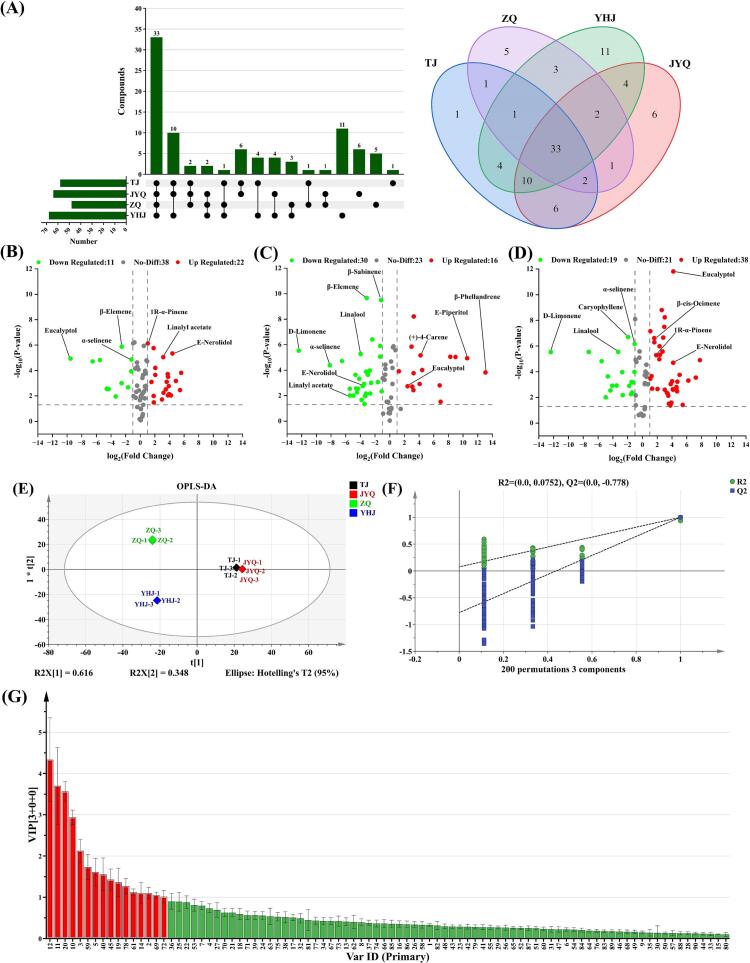
Multivariate analysis of VOCs in four ZAEOs. (A) Upset plot and Venn diagram of the total number of VOCs. (B) Volcano plot for comparison of JYQEO vs. TJEO. (C) Volcano plot for comparison of ZQEO vs. TJEO. (D) Volcano plot for comparison of YHJEO vs. TJEO. (E) OPLS-DA score plot of VOCs. (F) This graph is cross-validated by 200 permutation tests. (G) VIP scores, with red corresponding to compounds with VIP ≥ 1 and green indicating compounds with VIP < 1. (For interpretation of the references to color in this figure legend, the reader is referred to the web version of this article.)

As demonstrated in Fig. 3A, the intersection of volatile compounds among four ZAEOs was visually presented through Upset plot and Venn diagram. The number of compounds in the four ZAEOs ranged from 48 to 68, with 33 common components identified. Notably, YHJEO, JYQEO, ZQEO, and TJEO exhibited 11, 6, 5, and 1 unique components respectively, while YHJEO displayed the highest number of distinctive constituents, demonstrating the most significant compositional divergence from the other ZAEOs. To investigate the principal differences between cultivars and better understand the variation in VOC profiles among the four cultivars, volatile components were screened based on the criteria of fold change (FC) ≥ 2 or ≤ 0.5. Volcano plots were employed to effectively visualize the differences in VOCs across four ZAEOs. In Figs. 3B–D, red and green dots represented compounds with statistically significant two-fold increases and decreases, respectively. The analysis revealed 33 significantly differential VOCs between JYQEO and TJEO (22 upregulated and 11 downregulated, Fig. 3B), 46 differential VOCs between ZQEO and TJEO (16 upregulated and 30 downregulated, Fig. 3C), and the most substantial variation (57 differential VOCs) between YHJEO and TJEO (38 upregulated and 19 downregulated, Fig. 3D). The predominant differential components showing upregulation or downregulation between cultivars were identified as eucalyptol, β-phellandrene, linalool, d-limonene, β-elemene, β-myrcene, linalyl acetate, *E*-nerolidol, 1R-α-pinene, germacrene D, and α-selinene. These VOCs may serve as potential differential markers between groups, with their compositional and quantitative variations being crucial for modifying the flavor characteristics of ZAEO (Xing et al., 2025; Zhao et al., 2025). The unique combination patterns of these VOCs may be the key contributing factors underlying the distinctive sensory characteristics of the four ZAEOs.

#### 3.2.3 OPLS-DA analysis

OPLS-DA, a novel multivariate statistical method for regression modeling with multiple independent variables, was employed to accurately identify key variables influencing the compositional differences among four ZAEOs. The score plot of the four samples demonstrated complete separation and excellent model fit parameters (*R*^2^X = 0.999, *R*^2^Y = 1, *Q*^2^ = 1) (Fig. 3E). The reliability of the model was further confirmed by *R*^2^ and *Q*^2^ values exceeding 0.5. Following 200 permutation tests, the intercept of the *Q*^2^ regression line with the vertical axis was below zero, indicating no overfitting and validating the model robustness (*R*^2^ = (0.0, 0.0752), *Q*^2^ = (0.0, −0.778)) (Fig. 3F). These results provided a robust foundation for identifying and analyzing the aroma profiles of four ZAEOs. VIP values were utilized to quantify the contribution of each OPLS-DA variable to classification. A VIP score ≥ 1 is generally considered indicative of inter-group variability, playing a critical role in differentiation (Feng et al., 2025). In this study, an OPLS-DA model integrated with statistically significant differences (*p* < 0.05) was developed to screen distinct aroma-active compounds based on variations in the percentage composition of VOCs across samples (Fig. 3G). Ultimately, 16 differential VOCs with VIP ≥ 1 and *p* < 0.05 were identified (Table S2), which can be used to distinguish four ZAEOs.

### 3.3 Spectroscopic characterization of ZAEO

Vibrational spectroscopy (IR/Raman) offers advantages such as rapid, non-destructive analysis and minimal sample preparation (Rodríguez-Solana et al., 2014). Recent studies have demonstrated its reliability in chemometrics-based discrimination of essential oil varieties, authentication, and adulteration detection (Cebi et al., 2021). Meanwhile, IR and Raman spectroscopy can verify fine molecular structures and reflect overall intermolecular interactions in the mixture, complementing GC–MS.

#### 3.3.1 FT-IR spectra

To characterize the chemical compositions and functional group signatures of TJEO, JYQEO, ZQEO, and YHJEO, FT-IR spectroscopy was employed. As shown in Fig. S2A, the FT-IR spectra of TJEO, JYQEO, ZQEO, and YHJEO revealed distinct functional group signatures, with characteristic absorption bands consistent with their major chemical constituents. TJEO and JYQEO exhibited highly similar spectral profiles. The intense absorption peaks at 3379–3395 cm^−1^, corresponding to O—H stretching vibrations of alcohols/phenols, and the prominent peaks at 2924–2963 cm^−1^, attributed to C—H stretching vibrations of alkanes, directly correlated with their shared high-content constituents, including linalool (>34 %), d-limonene (>21 %), and β-sabinene (>14 %). The strong peaks at 1645–1647 cm^−1^ were assigned to C=C stretching vibrations of monoterpenes such as β-myrcene and limonene. Weak signals in the 1727–1728 cm^−1^ region suggested trace carbonyl groups from esters or aldehydes, consistent with the low-content ester and aldehyde compounds detected via GC–MS. Notably, the intense absorption peaks at 887–918 cm^−1^, characteristic of C—H out-of-plane bending vibrations in trisubstituted alkenes, matched the cyclic structure of β-sabinene (Zou et al., 2025). In contrast, ZQEO and YHJEO displayed distinct spectral patterns. ZQEO lacked prominent hydroxyl peaks above 3000 cm^−1^ but showed enhanced signals at 1465 cm^−1^ (C—H deformation vibrations of cyclic monoterpenes) and 1080 cm^−1^ (C—O stretching vibrations), aligning with the chemical properties of its major components, β-phellandrene (33.9 %) and eucalyptol (15.8 %). For YHJEO, the exceptionally high eucalyptol content (53.3 %) was evidenced by a characteristic strong peak at 841 cm^−1^ (ring vibrations of eucalyptol) and intensified absorption at 1080 cm^−1^ (C–O–C stretching vibrations of ether bonds). Both ZQEO and YHJEO lacked strong absorption near 1645 cm^−1^, indicating minimal conjugated double-bond structures, which corroborated their significantly lower limonene and myrcene levels compared to TJEO/JYQEO. These spectral distinctions demonstrated that FT-IR may effectively differentiate four ZAEOs through functional group vibrational features, even when major components overlap between samples, highlighting its discriminative capability.

#### 3.3.2 Raman spectra

The Raman spectral analysis revealed distinct vibrational patterns corresponding to the chemical composition variations among four ZAEOs (Fig. S2B). TJEO and JYQEO exhibited remarkably similar spectral profiles, characterized by intense peaks at 2915 cm^−1^ (C—H stretching in methyl/methylene groups) and 1641 cm^−1^ (C=C stretching vibrations), consistent with their shared dominant components linalool (34.4–35.5 %) and d-limonene (21.1–21.7 %) (Cebi et al., 2021). The prominent 1676 cm^−1^ band in both oils likely corresponds to conjugated carbonyl groups present in minor constituents like germacrene D. Notably, the 1448 cm^−1^ peak (CH_2_/CH_3_ deformation) showed higher relative intensity in TJEO, possibly reflecting its elevated β-sabinene content (14.9 % vs 18.2 % in JYQEO). ZQEO displayed a unique spectral signature dominated by a 1636 cm^−1^ peak (attributed to the conjugated double bonds of β-phellandrene), which aligns with its high β-phellandrene content (33.9 %). The spectral shift to 2933 cm^−1^ (asymmetric CH₃ stretching) differentiates ZQEO from other samples, possibly due to its unique terpene profile containing 11.6 % β-myrcene. YHJEO's spectrum showed exceptional features including a dominant 2930 cm^−1^ peak and enhanced 544 cm^−1^ signal (C-O-C stretching in ethers), correlating with its exceptionally high eucalyptol content (53.3 %) and ether-class predominance (53.4 %). The reduced intensity of C=C stretching bands (1638 cm^−1^) in YHJEO compared to other samples confirmed its lower terpene content (31.5 % vs 55.2–70.2 % in others), consistent with GC–MS quantification.

### 3.4 Biological activities

#### 3.4.1 In vitro antioxidant activity

**Fig. 4:**
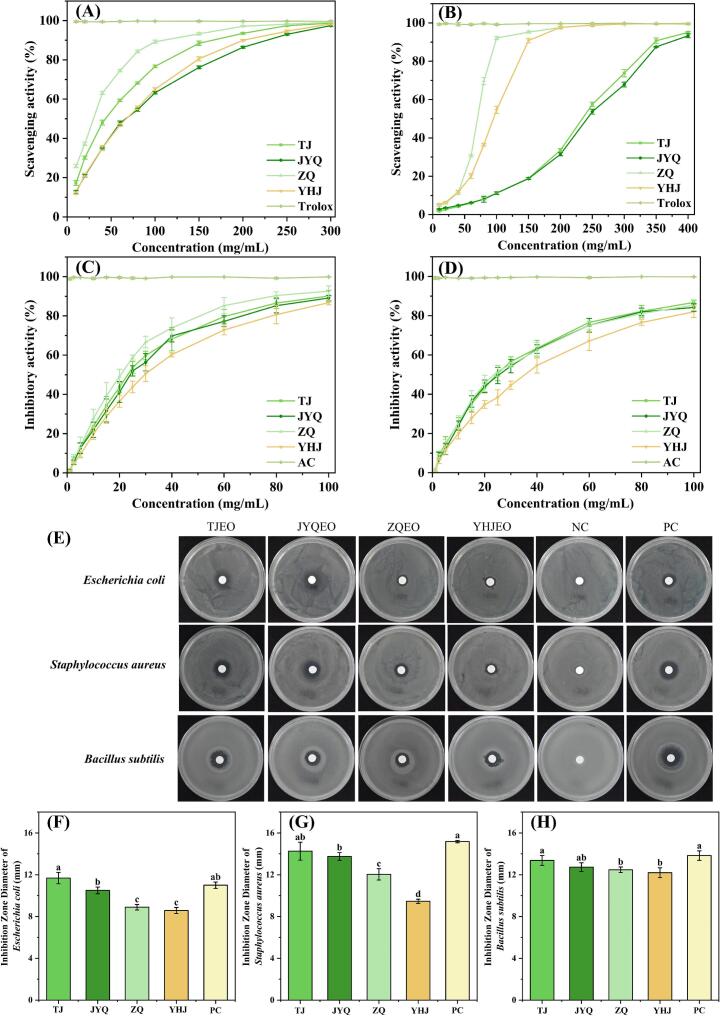
Comparison of biological activities among four ZAEOs. (A) ABTS radical scavenging activity. (B) DPPH radical scavenging activity. (C) α-Glucosidase inhibitory activity. (D) α-Amylase inhibitory activity. (E) Bacterial growth. (F) Inhibition zone diameters of *E. coli*. (G) Inhibition zone diameters of *S. aureus*. (H) Inhibition zone diameters of *B. subtilis*. Different letters indicate significant differences (*p* < 0.05). NC, negative control. PC, positive control.

The in vitro antioxidant activities of four ZAEOs were assessed through DPPH and ABTS assays. As shown in Fig. 4A, ZAEO exhibited concentration-dependent scavenging capacities against ABTS radicals. IC50 values of TJEO, JYQEO, ZQEO, YHJEO, and Trolox in ABTS radical scavenging were determined as 42.32 ± 0.93 mg/mL, 63.26 ± 0.87 mg/mL, 25.93 ± 0.56 mg/mL, and 62.12 ± 1.35 mg/mL, respectively (Fig. S3A). Fig. 4B demonstrated that ZAEO displayed relatively weaker but still concentration-dependent DPPH radical scavenging activities. The corresponding IC50 values for DPPH radical inhibition were 228.62 ± 1.05 mg/mL (TJEO), 240.31 ± 1.95 mg/mL (JYQEO), 66.25 ± 0.89 mg/mL (ZQEO), and 92.73 ± 2.42 mg/mL (YHJEO) (Fig. S3B). Notable variations were observed between ABTS and DPPH radical scavenging capabilities of four ZAEOs. This discrepancy may be attributed to the distinct reaction mechanisms of the two assay systems and the differential interactions between EO components and specific free radicals (Rumpf et al., 2023).

Generally, the antioxidant activity of EO is primarily associated with its volatile constituents (Ge et al., 2022; Zhang et al., 2017). ZQEO was primarily composed of β-phellandrene (33.9 %) and eucalyptol (15.8 %), whereas TJEO and JYQEO were dominated by linalool (34.4–35.5 %) and d-limonene (21.1–21.7 %). YHJEO contained eucalyptol (53.3 %) as its major constituent. Previous studies have demonstrated significant antioxidant potential of β-phellandrene and d-limonene, while the antioxidant mechanism of eucalyptol may involve electron transfer and free radical stabilization (Ge et al., 2022; Li, Liu, et al., 2022). Notably, the unique β-phellandrene content in ZQEO might contribute to its superior ABTS scavenging capacity, as this compound has been proven to effectively quench free radicals through a hydrogen donation mechanism. However, it should be emphasized that the overall antioxidant potential typically arises from synergistic interactions among multiple active compounds (Wang, Zhou, et al., 2024). The observed differences in antioxidant efficacy among the four ZAEOs likely reflect variations in their specific chemical compositions and the relative proportions of key compounds. For instance, the synergy between d-limonene and γ-terpinene significantly improves free radical scavenging efficiency (Li, Liu, et al., 2022). The present study showed that the inferior antioxidant activity of YHJEO is likely attributable to the absence of critical synergistic components compared to ZQEO, despite its high eucalyptol content of 53.3 %. This comparative analysis underscores ZQEO as a promising antioxidant source for applications. Its superior performance may be attributed to the dominant role of β-phellandrene, coupled with the synergistic contributions from multiple monoterpenes. These findings provide valuable insights into the design of efficient antioxidants, emphasizing the importance of synergistic interactions among bioactive constituents in these four ZAEOs.

#### 3.4.2 In vitro antidiabetic activity

EO have demonstrated potential in managing obesity, overweight conditions, dyslipidemia, and diabetes by modulating metabolic pathways and influencing physiological processes. Their complex composition, rich in terpenes and terpenoids, contributes to diverse bioactivities (Raut & Karuppayil, 2014). As depicted in Fig. 4, the enzymatic inhibitory activities of four ZAEOs were systematically evaluated. The investigated EOs exhibited dose-dependent inhibitory effects on both α-glucosidase and α-amylase (Fig. 4C and D), with their potency quantified by IC50 values (Figs. S3C and S3D). While ZAEOs demonstrated reduced efficacy against α-glucosidase relative to acarbose, TJEO, JYQEO, ZQEO, and YHJEO still exhibited moderate inhibitory activity, with IC50 values of 23.25 ± 1.44 mg/mL, 24.45 ± 1.18 mg/mL, 20.66 ± 1.26 mg/mL, and 29.76 ± 1.96 mg/mL, respectively (Fig. S3C). Similarly, α-amylase inhibition activities by four ZAEOs were less potent than that of acarbose. TJEO, JYQEO, and ZQEO exhibited comparable α-amylase inhibitory activity, yielding IC50 values of 24.21 ± 2.18 mg/mL, 25.55 ± 1.82 mg/mL, and 23.85 ± 1.36 mg/mL, respectively (Fig. S3D). By contrast, YHJEO exhibited the weakest inhibitory effect, with an IC50 value of 35.88 ± 3.25 mg/mL. These findings suggested that ZAEOs, particularly ZQEO, possess notable enzyme inhibitory potential, warranting further exploration for applications in functional foods or pharmaceutical development. These findings align with previous studies discussing the in vitro hypoglycemic effects of EO through digestive enzyme inhibition (Liang et al., 2023). In the present study, TJEO, JYQEO, ZQEO, and YHJEO, showed α-glucosidase inhibition rates of 90.1 %, 89.1 %, 92.6 %, and 86.8 % (Fig. 4C), respectively, while their α-amylase inhibition rates reached 86.8 %, 84.2 %, 85.4 %, and 82.0 % (Fig. 4D). Among these, ZQEO displayed the strongest α-glucosidase inhibitory activity, whereas TJEO, JYQEO, and ZQEO exhibited comparable α-amylase inhibition, with YHJEO being the least effective. Component analysis indicated that the high levels of β-phellandrene and eucalyptol in ZQEO likely contribute to its pronounced antidiabetic activity. However, although JYQEO and TJEO contain elevated concentrations of d-limonene and linalool, compounds known to regulate glucose and lipid metabolism (Radünz et al., 2021), the individual contributions of these components to the overall inhibitory effects remain unclear. This suggested that the enzyme-inhibitory effects of ZAEOs may arise from synergistic interactions among terpenoids (e.g., monoterpenes, oxygenated monoterpenes, and sesquiterpenes) rather than isolated actions of single compounds. These findings suggested their potential roles in moderating postprandial hyperglycemia. However, further validations through compound isolation and combinatorial experiments will be required to elucidate specific mechanisms.

#### 3.4.3 Antimicrobial activity

Bacterial growth is a critical factor in food spoilage, and foodborne infections can severely impair human physiological functions and health (Snyder et al., 2024). EO, owing to their broad-spectrum antimicrobial properties, exhibit significant application potential in the fields of food preservation and pharmaceuticals. As shown in Table S3, this study systematically evaluated the MICs and MBCs of four ZAEOs, including TJEO, JYQEO, ZQEO, and YHJEO, against *Escherichia coli* (*E. coli*), *Staphylococcus aureus* (*S. aureus*), and *Bacillus subtilis* (*B. subtilis*). ZAEOs exhibited significant antibacterial effects against *S. aureus, B. subtilis* and *E. coli* (Fig. 4E), with TJEO showing the strongest activity, followed by JYQEO, while YHJEO demonstrated the weakest efficacy. Specifically, the MIC values of TJEO against *E. coli*, *S. aureus*, and *B. subtilis* were 6.25 mg/mL, 3.125 mg/mL, and 3.125 mg/mL, respectively (Table S3). The agar diffusion assay further validated the above results. TJEO produced the largest inhibition zones against the three bacterial strains (11.68 ± 0.54 mm, 14.26 ± 0.86 mm, and 13.37 ± 0.47 mm, Figs. 4F–H), comparable to the positive control (0.25 mg/mL cefuroxime sodium).

This study demonstrated that ZAEO exhibit stronger antimicrobial activity against Gram-positive bacteria than Gram-negative bacteria, aligning with observations by Lou et al. (2024). This selectivity likely arised from structural differences in bacterial cell walls. Specifically, Gram-positive bacteria lack an outer membrane and possess a thick peptidoglycan layer, whereas Gram-negative bacteria have a thin peptidoglycan layer enclosed by a lipopolysaccharide-rich outer membrane (Govardhan Singh et al., 2013). The reduced susceptibility of Gram-negative bacteria may be linked to the limited permeability of their outer membrane to antimicrobial agents. The antimicrobial mechanisms of EOs are mediated through three principal modes of action. Membrane disruption, wherein hydrophobic constituents destabilize bacterial membranes, inducing ion leakage, depolarization, and compromising proton gradient maintenance. Enzyme inhibition, where key compounds such as α-pinene and β-myrcene disrupt enzymatic activity by interfering with hydrogen bonding at catalytic sites. Synergistic interactions, where secondary components (e.g., *Origanum vulgare* alcohol) enhance antimicrobial efficacy through additive or synergistic pathways (Li, Erhunmwunsee, et al., 2022). Component analysis indicated that the superior antibacterial activity of TJEO was strongly associated with its high content of linalool (34.4 %) and d-limonene (21.7 %) (Fig. S4). Previous studies have identified limonene and γ-terpinene as primary antibacterial constituents of EOs, while β-ocimene, β-pinene, and β-elemene also exhibit bactericidal properties (Tang, Zhang, et al., 2023). Notably, TJEO demonstrated significantly stronger selective antibacterial activity against *E. coli*, *S. aureus*, and *B. subtilis* compared to the other three ZAEOs, likely due to its unique synergistic composition. These findings emphasized that the antibacterial efficacy of ZAEOs depends not only on the concentration of individual components but also on synergistic interactions among constituents.

#### 3.4.4 Relationship between VOCs and biological activity

**Fig. 5:**
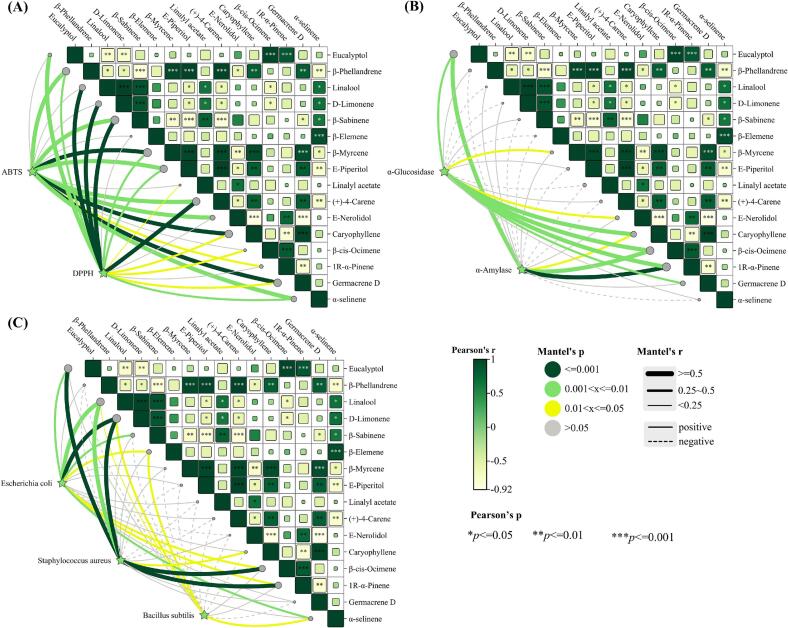
Mantel test analysis of correlations between key VOCs (VIP > 1.5, *p* < 0.05) and bioactivity metrics. (A) Correlation between differential VOCs and ABTS and DPPH. (B) Correlation between differential VOCs and α-glucosidase and α-amylase. (C) Correlation between differential VOCs and *E. coli*, *S. aureus*, and *B. subtilis*.

Multiple studies have demonstrated that the VOC profile in EO and its synergistic interactions significantly influence bioactivity intensity (Hou et al., 2022). The Mantel test, a non-parametric statistical method, was employed to assess correlations between independent distance matrices. This study implemented Mantel tests to examine relationships among antioxidant activity, antidiabetic activity, antibacterial activity, and volatile components of ZAEOs (Fig. 5). Line thickness illustrated correlation strength, while line color indicated statistical significance. Color blocks represented intra-group correlations. The statistical analysis revealed significant correlations between ZAEOs components and antioxidant activity. As illustrated in Fig. 5A, terpenoid constituents such as β-phellandrene, β-sabinene, and α-selinene exhibited strong positive correlations (*r* > 0.5, *p* < 0.05) in both ABTS and DPPH radical scavenging assays. Notably, linalool and d-limonene demonstrated exceptional performance in DPPH radical scavenging capabilities (*r* > 0.9, *p* < 0.01), which may be attributed to the electron-donating capacity of free phenolic hydroxyl groups and the radical trapping mechanism mediated by conjugated double-bond systems in their molecular structures. These findings aligned with previous studies on the antioxidant mechanisms of monoterpenes (Hou et al., 2022). Regarding antidiabetic activity, eucalyptol, caryophyllene, β-cis-ocimene, and 1R-α-pinene showed significant inhibitory effects on both α-glucosidase and α-amylase (r > 0.5, *p* < 0.05) (Fig. 5B). Of particular interest was the synergistic inhibition of β-cis-ocimene on dual enzymes. Its branched-chain structure likely obstructs substrate binding at the enzyme active site via steric hindrance, a phenomenon previously reported in hypoglycemic studies of oregano EO (Radünz et al., 2021). Antibacterial activity analysis revealed distinct responses across bacterial strains. For *E. coli*, linalool and d-limonene exhibited the most pronounced synergistic antibacterial effects (*r* > 0.8, *p* < 0.01) (Fig. 5C), likely due to their amphiphilic structures disrupting cell membrane integrity (Falleh et al., 2020). In contrast, *S. aureus* showed broader synergistic susceptibility to multiple monoterpenes (e.g., β-myrcene, β-phellandrene) and sesquiterpenes (e.g., caryophyllene, α-selinene). This discrepancy may stem from the permeability barrier posed by the thicker peptidoglycan layer in Gram-positive bacteria against hydrophobic components. These findings not only validated the efficacy of “component-activity” correlation analysis but also elucidated the multi-target functional characteristics of specific terpenoids in ZAEO.

### 3.5 Network pharmacology analysis

Network pharmacology is a key approach for deciphering the polypharmacological mechanisms of natural products by mapping compound-target-pathway interactions (Yu et al., 2024). It serves as an effective methodology to screen key targets and biological pathways underlying the antibacterial activity of ZAEO against *E. coli* and *S. aureus*. Topological analysis identified central hub proteins within the core interaction network, while functional enrichment elucidated the associated biological processes and pathways.

#### 3.5.1 Targets and network in *Escherichia coli*

**Fig. 6:**
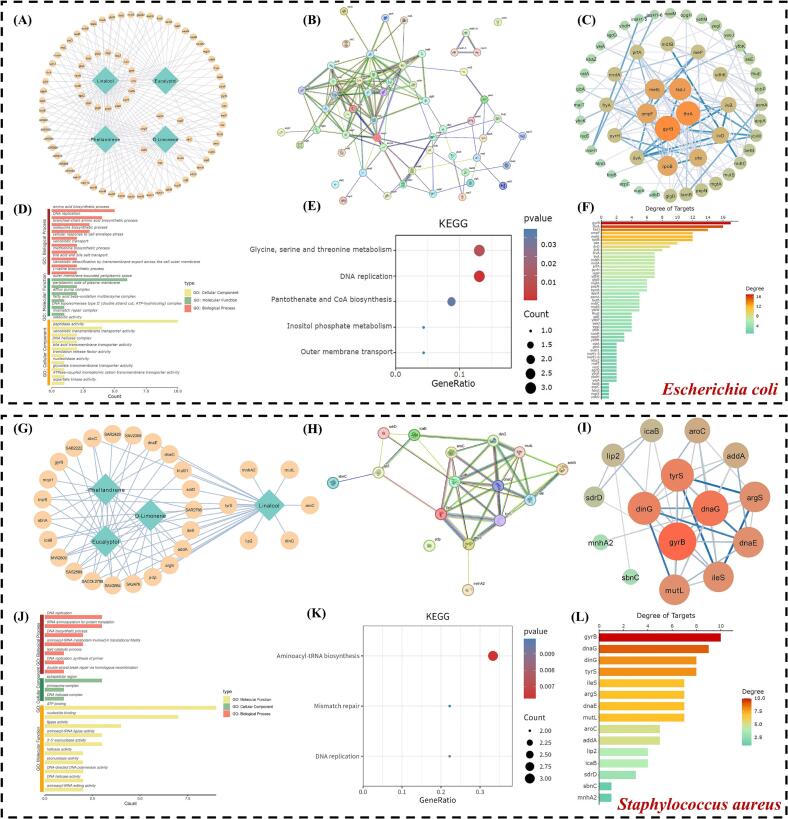
Network pharmacology analysis of the anti-*E. coli* (A–F) and anti-*S. aureus* (G–L) properties of four major ZAEO components. (A, G) Compound-target interaction network. (B, H) Intersecting targets network. (C, I) Core targets PPI network. (D, J) GO enrichment analysis. (E, K) KEGG pathway enrichment analysis. (F, L) Target degree ranking.

DeepPurpose prediction identified 83 *E. coli* protein targets exhibiting high-affinity binding (−log(Kd) ≥ 5.5) to the four major ZAEO components (Fig. 6A). PPI analysis generated a core network of 53 functionally cohesive interacting proteins (Figs. 6B–C). Topological assessment identified DNA gyrase subunit B (*gyrB*) and outer membrane porin F (*ompF*) as top-degree hubs (Fig. 6F). The selection of these targets is strongly supported by their established biological roles and therapeutic relevance: DNA gyrase (encoded by *gyrB*; PDB ID: 1KZN) plays a pivotal role in maintaining DNA structural stability, directly participating in critical processes such as DNA replication and transcription. Critically, the absence of its B subunit in mammals positions this enzyme as a key target for antimicrobial development (Weidlich & Klostermeier, 2020). OmpF (encoded by *ompF*; PDB ID: 2OMF) facilitates transmembrane transport of hydrophilic small molecules through its β-barrel tertiary structure. Its regulatory function in pore size critically influences bacterial nutrient uptake and antibiotic permeability, with downregulation strongly correlated to multidrug resistance (Choi & Lee, 2019). Their high network centrality and fundamental roles in essential bacterial processes (DNA topology maintenance and membrane integrity/permeability) strongly justify their selection as core targets for molecular docking validation of ZAEO's anti-*E. coli* activity.

Functional enrichment analysis (GO and KEGG) further elucidated the network's antibacterial relevance (Fig. 6D**–**E), corroborating the significance of *gyrB* and *ompF*. Key enriched biological processes included DNA replication (directly involving GyrB), branched-chain amino acid biosynthesis (isoleucine, valine), xenobiotic detoxification, and cell envelope stress response. Molecular functions encompassed catalytic activity, transmembrane transporter activity (e.g., bile acid, xenobiotic), and critical complexes such as DNA topoisomerase type II (GyrB) and efflux pumps, supporting *ompF*'s role in membrane integrity (Zhou et al., 2023). Cellular component enrichment localized to the outer membrane and periplasmic space, consistent with OmpF's function. KEGG pathway analysis enriched Valine/leucine/isoleucine biosynthesis, DNA replication, and notably, outer membrane transport (directly involving OmpF). Collectively, the topological dominance of *gyrB* and *ompF*, coupled with their functional links to indispensable processes (DNA topology maintenance and membrane permeability), positions them as primary mechanistic targets for investigation. Their well-characterized crystal structures (PDB: 1KZN, 2OMF) further enable molecular docking studies to validate ZAEO binding and elucidate its anti-*E. coli* mechanism of action.

#### 3.5.2 Targets and network in *Staphylococcus aureus*

Analysis of the *S. aureus* target network (29 high-affinity proteins, Fig. 6G) identified DNA gyrase subunit B (*gyrB*) and tyrosyl-tRNA synthetase (*tyrS*) as the dominant topological hubs within the core PPI network (15 proteins, Figs. 6H–I), ranking first and fourth respectively in degree centrality (Fig. 6L). These hubs represent mechanistically compelling targets with significant therapeutic potential. Consistent with its essential role in *E. coli* (Section 3.5.1), DNA gyrase (encoded by gyrB; PDB ID: 2XCT) represents a validated, high-priority antibacterial target in *S. aureus*. Tyrosyl-tRNA synthetase (TyrS; PDB ID: 1JIJ) is essential during the aminoacylation phase of protein synthesis. Inhibition of aminoacyl-tRNA synthetases like TyrS effectively disrupts bacterial metabolism, making them ideal targets for novel antimicrobial agents (Wang, Leng, et al., 2024). Their high network connectivity and distinct, essential functions strongly support their selection for molecular docking against *S. aureus*.

Functional enrichment further validated the network's biological significance (Figs. 6J**–**K). GO analysis highlighted essential processes: DNA replication, DNA biosynthesis, double-strand break repair (all involving GyrB), and tRNA aminoacylation for protein translation (directly mediated by TyrS). Molecular functions were enriched for ATP binding, DNA helicase activity, aminoacyl-tRNA ligase activity (TyrS), and 3′-5′ exonuclease activity. KEGG pathway analysis confirmed DNA replication (GyrB-associated) and Aminoacyl-tRNA biosynthesis (TyrS-associated) as key enriched pathways (Fig. 6K), directly reinforcing the centrality of these targets. In summary, the topological prominence of *gyrB* and *tyrS* within the PPI network, coupled with their distinct and complementary roles in DNA topology control (GyrB) and protein synthesis (TyrS), identifies them as highly relevant and mechanistically compelling targets for validation. Their available crystal structures (PDB: 2XCT, 1JIJ) provide a solid structural basis for molecular docking to validate ZAEO binding and investigate its anti-*S. aureus* mechanism of action.

### 3.6 Molecular docking

**Fig. 7:**
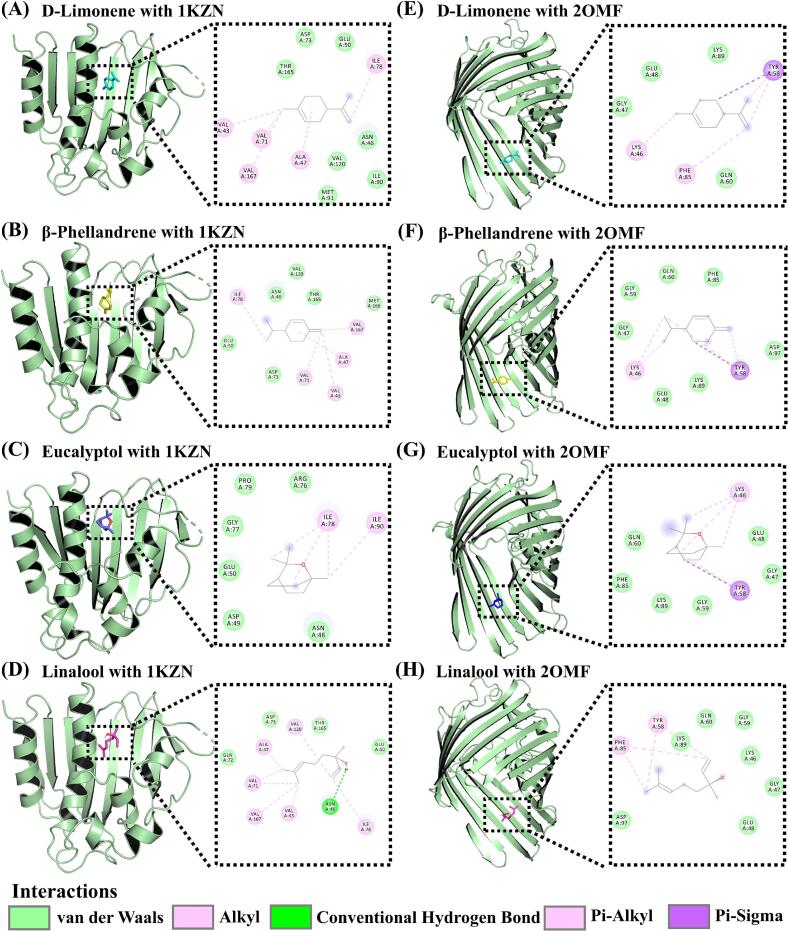
Molecular docking of d-limonene (A, E), β-phellandrene (B, F), eucalyptol (C, G), and linalool (D, H) with the active sites of *Escherichia coli* DNA gyrase (PDB ID: 1KZN) and outer membrane protein OmpF (PDB ID: 2OMF). From left to right, the panels show the 3D structure and 2D interaction diagram. In 3D views, the protein is depicted as a light green cartoon model. Ligands are displayed as stick models: d-limonene (cyan), β-phellandrene (yellow), eucalyptol (blue), and linalool (magenta). In 2D diagrams, hydrogen bonds are denoted by green dashed lines, while hydrophobic interactions are indicated by pink and purple dashed lines. (For interpretation of the references to color in this figure legend, the reader is referred to the web version of this article.)

**Fig. 8:**
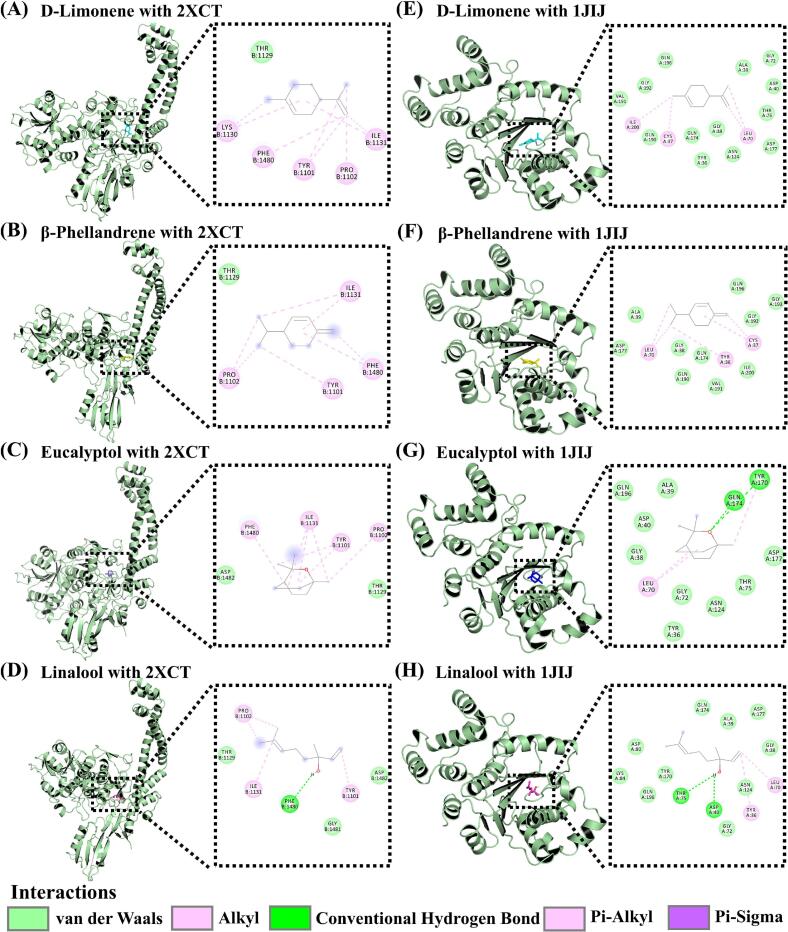
Molecular docking of d-limonene (A, E), β-phellandrene (B, F), eucalyptol (C, G), and linalool (D, H) with the active sites of *Staphylococcus aureus* DNA gyrase (PDB ID: 2XCT) and tyrosyl-tRNA synthetase (PDB ID: 1JIJ). From left to right, the panels show the 3D structure and 2D interaction diagram. In 3D views, the protein is represented as a light green cartoon model. Ligands are shown as stick models: d-limonene (cyan), β-phellandrene (yellow), eucalyptol (blue), and linalool (magenta). In 2D diagrams, hydrogen bonds are marked by green dashed lines, while hydrophobic interactions are indicated by pink and purple dashed lines. (For interpretation of the references to color in this figure legend, the reader is referred to the web version of this article.)

Molecular docking analyses (Table S4) of four ZAEO components, including d-limonene, β-phellandrene, eucalyptol, and linalool, revealed variations in binding affinity that aligned with their antibacterial activity trends. As shown in Fig. 7, Fig. 8, and Fig. S5, d-limonene and β-phellandrene exhibited strong affinities for all four target proteins, with binding energies below −5.0 kcal/mol. Generally, binding energies <0 kcal/mol indicated spontaneous ligand-receptor binding without external energy input, values < −5.0 kcal/mol suggest favorable binding, and values < −7.0 kcal/mol denoted robust interactions. Lower binding energies correlate with superior docking performance (Noshad et al., 2022). Interaction analyses demonstrated that these terpenoids bind by occupying protein cavities and forming hydrogen bonds or hydrophobic interactions with critical residues. For instance, linalool, a major component of TJEO and JYQEO, established hydrogen bonds with ASP40 and THR75 of 1JIJ (binding energy: −4.9 kcal/mol), potentially inhibiting protein synthesis by disrupting aminoacylation. In contrast, d-limonene interacted with CYS37 and ILE200 of 1JIJ via hydrophobic forces (binding energy: −5.7 kcal/mol), likely destabilizing the enzymatic active site (Wang, Leng, et al., 2024). This dual mechanism explained TJEO's superior antibacterial efficacy against *S. aureus*, where synergistic hydrogen bonding and hydrophobic interactions enhance complex stability. While β-phellandrene demonstrated binding energies comparable to d-limonene against targets 1KZN and 2OMF (−5.7 and − 5.0 kcal/mol, respectively), the overall antimicrobial activity of ZQEO (containing 33.9 % β-phellandrene) remained inferior to that of TJEO. This discrepancy might be attributed to the effective antibacterial synergy between d-limonene and linalool, underscoring the critical role of multicomponent cooperative interactions in efficacy enhancement (Hao et al., 2022). Eucalyptol (53.3 % in YHJEO) displayed the weakest binding across targets (−4.6 to −4.7 kcal/mol), forming limited hydrophobic interactions with ILE78/ILE90 of 1KZN, consistent with its poor antibacterial performance. All tested ligands exhibited lower binding energies for *S. aureus* targets (2XCT and 1JIJ: −4.3 to −5.7 kcal/mol) compared to *E. coli* targets (e.g., 2OMF), possibly due to better compatibility between terpenoids and the hydrophobic pockets characteristic of Gram-positive enzymes. These observations supported the mechanism where component concentrations and target-specific binding patterns jointly determine antimicrobial efficacy (Kačániová et al., 2025).

Collectively, the antimicrobial efficacy of ZAEO may be attributed to the key roles of d-limonene and β-phellandrene, as well as the synergistic effects among multiple components. These compounds bind to DNA gyrase and tyrosyl-tRNA synthetase, thereby disrupting genetic material replication and expression. Simultaneously, they enhance membrane permeability in *E. coli* through interactions with OmpF. This multi-target mechanism of action, combined with multi-component synergistic interactions, provided valuable insights for the development of novel antimicrobial agents using ZAEO.

## 4 Conclusions

This study comprehensively examined the chemical composition and bioactive properties of EO derived from four *Z. armatum* cultivars. The results revealed significant antioxidant, antibacterial, and antidiabetic activities in these ZAEOs, with marked cultivar-dependent variations. GC–MS analysis detected approximately 90 VOCs, highlighting distinct differences in VOC profiles and concentrations across the four ZAEOs. FTIR and Raman spectroscopy further confirmed the chemical heterogeneity among them. The biological activities of ZAEO were predominantly ascribed to differential component concentrations and synergistic interactions among constituents. Notably, ZQEO demonstrated superior antioxidant and antidiabetic capacities, while TJEO exhibited the highest antibacterial efficacy. Network pharmacology and molecular docking simulations identified mechanistically significant targets in both *E. coli* and *S. aureus*, which are crucially involved in DNA replication and cellular membrane function. Specifically, these analyses predicted that d-limonene and β-phellandrene exert antibacterial effects by interfering with genetic material replication and modulating cellular membrane permeability, respectively targeting the identified pathways. The present study offered valuable insights into the distinct chemical compositions and bioactivities of four ZAEOs, suggesting that ZAEOs could serve as natural, effective agents with high safety for food, agricultural, and nutraceutical industries. However, further studies on extraction, purification, and mechanisms of ZAEOs are essential to confirm their safety and beneficial properties, which will be crucial for realizing their potential utility in the food industry.

## CRediT authorship contribution statement

**Shao-jun Fan:** Writing – review & editing, Writing – original draft, Software, Methodology, Investigation, Formal analysis, Data curation. **Wen-zhang Qian:** Writing – review & editing, Visualization, Methodology, Formal analysis. **Yi-xiao Xiao:** Writing – review & editing, Visualization, Investigation. **Yun-yi Hu:** Writing – review & editing, Visualization, Investigation. **Meng-lin Jiang:** Writing – review & editing, Investigation, Data curation. **Ji-cheng Chen:** Writing – review & editing, Supervision. **Dan-ju Zhang:** Writing – review & editing, Supervision. **Shun Gao:** Writing – review & editing, Writing – original draft, Supervision, Project administration, Funding acquisition, Conceptualization.

## Declaration of competing interest

The authors declare that they have no known competing financial interests or personal relationships that could have appeared to influence the work reported in this paper.

## Data Availability

Data will be made available on request.

## Appendix A Supplementary data

Supplementary material: Supplementary Figures and Tables Image 1

## References

[bb0005] Akachoud O., Bouamama H., Laruelle F., Facon N., Broudi E.L., Houssayni S., Qaddoury A. (2024). The developmental stage and arbuscular mycorrhizal symbiosis influence the essential oil yield, chemical profile, and biological activities in *Thymus pallidus*, *T. Satureioides*, and *Lavandula dentata*. Industrial Crops and Products.

[bb0010] Asbahani A.E., Miladi K., Badri W., Sala M., Addi E.H.A., Casabianca H., Elaissari A. (2015). Essential oils: From extraction to encapsulation. International Journal of Pharmaceutics.

[bb0015] Benmoussa H., Béchohra I., He S., Elfalleh W., Chawech R. (2023). Optimization of sonohydrodistillation and microwave assisted hydrodistillation by response surface methodology for extraction of essential oils from *Cinnamomum cassia* barks. Industrial Crops and Products.

[bb0020] Bizzo H.R., Brilhante N.S., Nolvachai Y., Marriott P.J. (2023). Use and abuse of retention indices in gas chromatography. Journal of Chromatography A.

[bb0025] Cebi N., Arici M., Sagdic O. (2021). The famous Turkish rose essential oil: Characterization and authenticity monitoring by FTIR, Raman and GC–MS techniques combined with chemometrics. Food Chemistry.

[bb0030] Cheng J., Ke J., Hou X., Li S., Luo Q., Shen G., Zhang Z. (2021). Changes in qualities of dried *Zanthoxylum armatum* DC. At different storage methods. Food Packaging and Shelf Life.

[bb0035] Choi U., Lee C.-R. (2019). Distinct roles of outer membrane porins in antibiotic resistance and membrane integrity in *Escherichia coli*. Frontiers in Microbiology.

[bb0040] Falleh H., Ben Jemaa M., Saada M., Ksouri R. (2020). Essential oils: A promising eco-friendly food preservative. Food Chemistry.

[bb0045] Feng S., Li P., Li Y., Xiao X., Chen X., Leng H., Liang H., Zhou L., Chen T., Ding C. (2025). Volatile profiles and characteristic odorants in *Camellia* seeds with different heat pretreatments. Food Chemistry.

[bb0050] Ge X., Liang Q., Long Y., Shen H., Zhang Q., Sun Z., Li W. (2022). Assessment of fresh *Alpinia galanga* (*A. Galanga*) drying techniques for the chemical composition of essential oil and its antioxidant and biological activity. Food Chemistry.

[bb0055] Govardhan Singh R.S., Negi P.S., Radha C. (2013). Phenolic composition, antioxidant and antimicrobial activities of free and bound phenolic extracts of *Moringa oleifera* seed flour. Journal of Functional Foods.

[bb0060] Hao Y., Kang J., Yang R., Li H., Cui H., Bai H., Tsitsilin A., Li J., Shi L. (2022). Multidimensional exploration of essential oils generated via eight oregano cultivars: Compositions, chemodiversities, and antibacterial capacities. Food Chemistry.

[bb0065] Hou T., Sana S.S., Li H., Xing Y., Nanda A., Netala V.R., Zhang Z. (2022). Essential oils and its antibacterial, antifungal and anti-oxidant activity applications: A review. Food Bioscience.

[bb0070] Huang D., Chen X., Tan R., Wang H., Jiao L., Tang H., Zong Q., Mao Y. (2024). A comprehensive metabolomics analysis of volatile and non-volatile compounds in matcha processed from different tea varieties. Food Chemistry: X.

[bb0075] Huang K., Fu T., Glass L.M., Zitnik M., Xiao C., Sun J. (2021). DeepPurpose: A deep learning library for drug–target interaction prediction. Bioinformatics.

[bb0080] Jaradat N., Al-Maharik N., Abdallah S., Shawahna R., Mousa A., Qtishat A. (2020). *Nepeta curviflora* essential oil: Phytochemical composition, antioxidant, anti-proliferative and anti-migratory efficacy against cervical cancer cells, and α-glucosidase, α-amylase and porcine pancreatic lipase inhibitory activities. Industrial Crops and Products.

[bb0085] Kačániová M., Vukic M.D., Vukovic N.L., Terentjeva M., Ban Z., Li L., Garzoli S. (2025). Chemical composition, antimicrobial, antibiofilm and insecticidal enhancing of *Eucalyptus citriodora* essential oil and its potential to shelf-life extension of pumpkin after inoculation of *Salmonella enterica*. Food Control.

[bb0090] Karalija E., Dahija S., Tarkowski P., Zeljković S.Ć. (2022). Influence of climate-related environmental stresses on economically important essential oils of Mediterranean *Salvia* sp. Frontiers in Plant Science.

[bb0095] Li Y., Erhunmwunsee F., Liu M., Yang K., Zheng W., Tian J. (2022). Antimicrobial mechanisms of spice essential oils and application in food industry. Food Chemistry.

[bb0100] Li Y., Liu S., Zhao C., Zhang Z., Nie D., Tang W., Li Y. (2022). The chemical composition and antibacterial and antioxidant activities of five *Citrus* essential oils. Molecules.

[bb0105] Liang S., Hu W., Cheng W., Zhang S., Zou R. (2023). *Zanthoxylum bungeanum* essential oil: Extraction and component analysis for α-glucosidase inhibitory activity and the underlying mechanism based on molecular docking. Applied Sciences.

[bb0110] Liu F., Kan Q., Feng K., Chen Y., Wen L., He B., Liu G. (2023). Process of *Zanthoxylum armatum* DC. Oil by a novel low-temperature continuous phase transition extraction: Evaluation of aroma, pungent compounds and quality. LWT.

[bb0115] Liu Y., Li Q., Yang W., Sun B., Zhou Y., Zheng Y., Yang W. (2020). Characterization of the potent odorants in *Zanthoxylum armatum* DC Prodr. Pericarp oil by application of gas chromatography–mass spectrometry–olfactometry and odor activity value. Food Chemistry.

[bb0120] Lou S., Ni X., Xiao W., Li Y., Gao Z. (2024). Physical stability, microstructure and antimicrobial properties of konjac glucomannan coatings enriched with *Litsea cubeba* essential oil nanoemulsion and its effect on citruses preservation. International Journal of Biological Macromolecules.

[bb0125] Meenu M., Padhan B., Patel M., Patel R., Xu B. (2023). Antibacterial activity of essential oils from different parts of plants against *Salmonella* and *Listeria* spp. Food Chemistry.

[bb0130] Noshad M., Alizadeh Behbahani B., Nikfarjam Z. (2022). Chemical composition, antibacterial activity and antioxidant activity of *Citrus* bergamia essential oil: Molecular docking simulations. Food Bioscience.

[bb0135] Qian Q., Zhuo Z., Peng Y., Xu D. (2024). Chemical composition variation in essential oil and their correlation with climate factors in Chinese prickly ash peels (*Zanthoxylum armatum* DC.) from different habitats. Molecules.

[bb0140] Radünz M., Mota Camargo T., Hackbart S., Dos H.C., Inchauspe Correa Alves P., Radünz A.L., da Rosa Zavareze E. (2021). Chemical composition and *in vitro* antioxidant and antihyperglycemic activities of clove, thyme, oregano, and sweet orange essential oils. LWT.

[bb0145] Raut J.S., Karuppayil S.M. (2014). A status review on the medicinal properties of essential oils. Industrial Crops and Products.

[bb0150] Rodríguez-Solana R., Daferera D.J., Mitsi C., Trigas P., Polissiou M., Tarantilis P.A. (2014). Comparative chemotype determination of *lamiaceae* plants by means of GC–MS, FT-IR, and dispersive-raman spectroscopic techniques and GC-FID quantification. Industrial Crops and Products.

[bb0155] Rumpf J., Burger R., Schulze M. (2023). Statistical evaluation of DPPH, ABTS, FRAP, and Folin-Ciocalteu assays to assess the antioxidant capacity of lignins. International Journal of Biological Macromolecules.

[bb0160] Shannon P., Markiel A., Ozier O., Baliga N.S., Wang J.T., Ramage D., Ideker T. (2003). Cytoscape: A software environment for integrated models of biomolecular interaction networks. Genome Research.

[bb0165] Shao Y., Liu X., Zhang Z., Wang P., Li K., Li C. (2023). Comparison and discrimination of the terpenoids in 48 species of huajiao according to variety and geographical origin by E-nose coupled with HS-SPME-GC-MS. Food Research International.

[bb0170] Singh Chouhan K.B., Tandey R., Sen K.K., Mehta R., Mandal V. (2019). Critical analysis of microwave hydrodiffusion and gravity as a green tool for extraction of essential oils: Time to replace traditional distillation. Trends in Food Science & Technology.

[bb0175] Snyder A.B., Martin N., Wiedmann M. (2024). Microbial food spoilage: Impact, causative agents and control strategies. Nature Reviews Microbiology.

[bb0180] Szklarczyk D., Gable A.L., Nastou K.C., Lyon D., Kirsch R., Pyysalo S., von Mering C. (2021). The STRING database in 2021: Customizable protein–protein networks, and functional characterization of user-uploaded gene/measurement sets. Nucleic Acids Research.

[bb0185] Tang N., Wu P., Cao Z., Liu Y., Zhang X., Lou J., Liu X., Hu Y., Sun X., Wang Q., Si S., Chen Z. (2023). A NAC transcription factor ZaNAC93 confers floral initiation, fruit development, and prickle formation in *Zanthoxylum armatum*. Plant Physiology and Biochemistry.

[bb0190] Tang W., Zhang Z., Nie D., Liu S., Li Y., Liu M., Li Y. (2023). Selective antibacterial activity of *Citrus Medica limonum* essential oil against *Escherichia coli* K99 and *lactobacillus acidophilus* and its antibacterial mechanism. LWT.

[bb0195] Wang H., Ren J., Zhou S., Duan Y., Zhu C., Chen C., Liu Z., Zheng Q., Xiang S., Xie Z., Wang X., Chai L., Ye J., Xu Q., Guo W., Deng X., Zhang F. (2024). Molecular regulation of oil gland development and biosynthesis of essential oils in *Citrus* spp. Science.

[bb0200] Wang H., Zhou X., Deng Y., Zhang R., Fu K., Huang J., Huang Q., Zeng C., Liu D., Wang W. (2024). Variations in volatile components and biological activities of essential oils from *Citrus aurantium* ‘changshanhuyou’ at different growth and ripening stages. Food Research International.

[bb0205] Wang W., Leng Z., Liu Q., Zhao J., Li S. (2024). Nano-emulsification of *Osmanthus* essential oil: Characterizations, stability and molecular interactions explaining antibacterial activity. Industrial Crops and Products.

[bb0210] Wang Y., Luo J., Hou X., Wu H., Li Q., Li S., Luo Q., Li M., Liu X., Shen G., Cheng A., Zhang Z. (2022). Physicochemical, antibacterial, and biodegradability properties of green Sichuan pepper (*Zanthoxylum armatum* DC.) essential oil incorporated starch films. LWT.

[bb0215] Wang Y., Wu Q., Jiang L., Ma D., Yu J., Cui Q., Zhang Z. (2025). Characteristic of essential oil in *zanthoxylum armatum* DC. Leaves and application in flavor oil. Food Chemistry.

[bb0220] Weidlich D., Klostermeier D. (2020). Functional interactions between gyrase subunits are optimized in a species-specific manner. Journal of Biological Chemistry.

[bb0225] Xia N., Wang J., Jia Y., Duan J., Wang X., Li J., Zhou P., Xie Y., Shi H., Zhao C., Zou J., Guo D., Shi Y., Li H., Wu Z., Yang M., Chang X., Sun J., Zhang X. (2025). Optimization of the process of extracting essential oil of rosemary by hydro distillation with different auxiliary methods. LWT.

[bb0230] Xing J., Yang C., Zhang L. (2025). Characterization of key flavor compounds in cinnamon bark oil extracts using principal component analysis. Food Research International.

[bb0235] Xing Z., Xu Y., Feng X., Gao C., Wu D., Cheng W., Meng L., Wang Z., Xu T., Tang X. (2024). Fabrication of cinnamon essential oil nanoemulsions with high antibacterial activities via microfluidization. Food Chemistry.

[bb0240] Yu W., Li X., Sun Q., Yi S., Zhang G., Chen L., Luo L. (2024). Metabolomics and network pharmacology reveal the mechanism of *Castanopsis* honey against *streptococcus pyogenes*. Food Chemistry.

[bb0245] Zhang L., Tu Z., Xie X., Wang H., Wang H., Wang Z., Lu Y. (2017). Jackfruit (*Artocarpus heterophyllus* lam.) peel: A better source of antioxidants and α-glucosidase inhibitors than pulp, flake and seed, and phytochemical profile by HPLC-QTOF-MS/MS. Food Chemistry.

[bb0250] Zhao Q., Li Z., Zhang K., Deng X., Wang G., Ye Z., Liu M., Chen J., Chen S., Ye X., Cheng H. (2025). Revealing the off-flavors in hydro-distilled essential oils of sweet orange (*Citrus sinensis*) by flavoromics strategy and computational simulation. Food Chemistry.

[bb0255] Zhao X., Wei Q., Wu H., Zhou W., Liu M., Yang L., Feng R., Li M. (2023). Changes in essential oils content, antioxidant capacity and secondary metabolism in different *Cinnamomum longepaniculatum* varieties. Industrial Crops and Products.

[bb0260] Zhou G., Wang Q., Wang Y., Wen X., Peng H., Peng R., Shi Q., Xie X., Li L. (2023). Outer membrane porins contribute to antimicrobial resistance in gram-negative bacteria. Microorganisms.

[bb0265] Zou X., Zhao S., Xu K., Liu K., Yan C., Zhang X., Chen J., Cheng Y., Fang C. (2025). Development and characterization of corn starch-based films enhanced with *Chlorella vulgaris* nanocellulose-stabilized Pickering emulsion of *Zanthoxylum bungeanum* essential oil for cherry tomato preservation. International Journal of Biological Macromolecules.

