# Supplementary material for: Comparative analysis of Zanthoxylum armatum essential oils from four cultivars: Chemical compositions, antioxidant activities, and antibacterial activities
Source: Food Chem X. 2025 Sep 6;30:103005. doi: 10.1016/j.fochx.2025.103005 (PMC12451176; doi:10.1016/j.fochx.2025.103005)
Supplement: Supplementary file 1 — Supplementary material: Supplementary Figures and Tables [file mmc1.docx]

**Supplementary material: Tables S1-S4 and Figures S1-S5**

**Table S1.** Information on introduction source sites and cultivation sites of four cultivars of *Zanthoxylum armatum*.

| **NO.** | **color** | **name** | **morphological features** | **introductio****n source site** | **coordinate** | **cultivation site/coordinate** | **Climate type** |
| --- | --- | --- | --- | --- | --- | --- | --- |
| TJ | Green | *Tengjiao* | 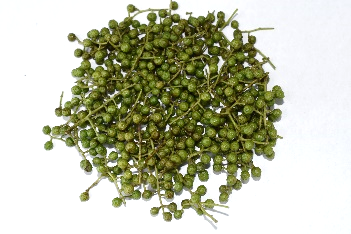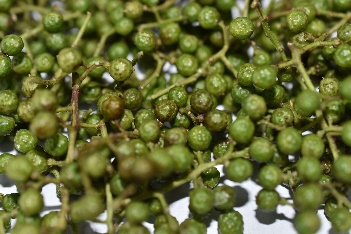 | Hongya County, Ya'an City, Sichuan Province, China | 29.35N, 106.33E | Shuangliu District, Chengdu City, Sichuan Province, China/30°22′10.09″N, 104°00′22.69″E | Subtropical monsoon climate |
| JYQ | Green | *Jiuyeqing* | 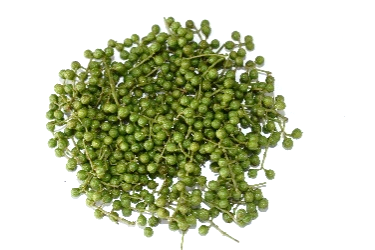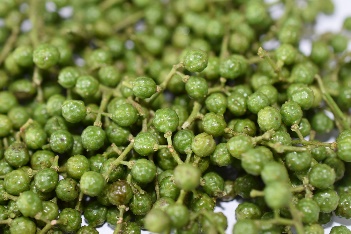 | Jiangjin District, Chongqing City, China | 29.92N, 103.37E |  | Subtropical monsoon climate |
| ZQ | Green | *Ziyang Qinghuajiao* | 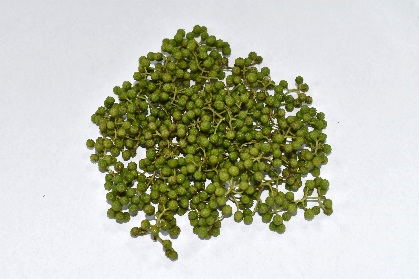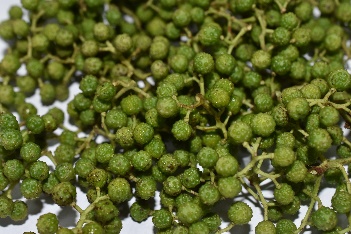 | Lezhi County Ziyang City, Sichuan Province, China | 30.38N, 104.99E |  | Subtropical monsoon climate |
| YHJ | Green | *Yehuajiao* | 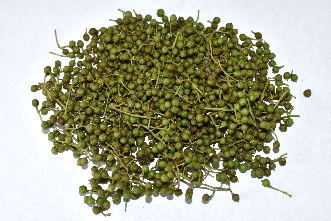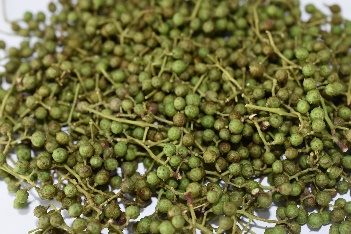 | Shuangliu District, Chengdu City, Sichuan Province, China | 30.37N, 104.01E |  | Subtropical monsoon climate |

*Tengjiao* (*Zanthoxylum armatum* 'Tengjiao', Approval No.: **Chuan S-SV-ZA-001-2014**) and *Jiuyeqing* (*Zanthoxylum armatum* 'Jiuyeqing', Approval No.: **Guo S-SV-ZA-020-2005**): Officially approved cultivars from Sichuan/Chongqing; distinct flavors (*Tengjiao*: mild pungency, fresh aroma, high oil; *Jiuyeqing*: intense pungency, pure fragrance). *Yehuajiao*: Wild resource ("Goushijiao"), wide adaptability; limited research due to low fruit/oil yield. *Ziyang Qinghuajiao*: Novel germplasm (this study); features larger fruit oil glands and higher essential oil yield; under development for elite cultivar registration.

**Table S2.** The differential VOCs among *Z. armatum* cultivars based on VIP > 1 and *p* < 0.05.

| No. | Compound name | CAS | Molecular formula | VIP | *Z. armatum* cultivars |
| --- | --- | --- | --- | --- | --- |
| 12 | Eucalyptol | 470-82-6 | C10H18O | 4.333 | YHJ, ZQ, TJ |
| 11 | β-Phellandrene | 555-10-2 | C10H16 | 3.700 | ZQ |
| 20 | Linalool | 78-70-6 | C10H18O | 3.563 | JYQ, TJ, YHJ, ZQ |
| 10 | D-Limonene | 5989-27-5 | C10H16 | 2.939 | TJ, JYQ |
| 3 | β-Sabinene | 3387-41-5 | C10H16 | 2.125 | JYQ, TJ, YHJ, ZQ |
| 59 | β-Elemene | 515-13-9 | C15H24 | 1.731 | TJ, YHJ, JYQ, ZQ |
| 5 | β-Myrcene | 123-35-3 | C10H16 | 1.609 | ZQ, TJ, JYQ YHJ |
| 40 | E-Piperitol | 16721-39-4 | C10H18O | 1.557 | ZQ |
| 45 | Linalyl acetate | 115-95-7 | C12H20O2 | 1.428 | JYQ, TJ, YHJ |
| 19 | (+)-4-Carene | 29050-33-7 | C10H16 | 1.358 | ZQ, YHJ, TJ, JYQ |
| 78 | E-Nerolidol | 40716-66-3 | C15H26O | 1.267 | JYQ, YHJ, TJ |
| 61 | Caryophyllene | 87-44-5 | C15H24 | 1.125 | ZQ, TJ, JYQ, YHJ |
| 14 | β-cis-Ocimene | 3338-55-4 | C10H16 | 1.104 | YHJ, ZQ, TJ, JYQ |
| 2 | 1R-α-Pinene | 7785-70-8 | C10H16 | 1.104 | YHJ, JYQ, ZQ, TJ |
| 69 | Germacrene D | 23986-74-5 | C15H24 | 1.052 | ZQ, TJ, JYQ, YHJ |
| 72 | α-selinene | 473-13-2 | C15H24 | 1.012 | TJ, YHJ, JYQ |

**Table S3.** MIC and MBC values (mg/mL) of essential oils from TJ, JYQ, ZQ, YHJ against *E. coli*, *S. aureus* and *B. subtilis*.

| EO | *E. coli* | | *S. aureus* | | *B. subtilis* | |
| --- | --- | --- | --- | --- | --- | --- |
|  | MIC (mg/mL) | MBC (mg/mL) | MIC (mg/mL) | MBC (mg/mL) | MIC (mg/mL) | MBC (mg/mL) |
| TJ | 6.25 | 12.5 | 3.125 | 3.125 | 3.125 | 6.25 |
| JYQ | 6.25 | 25 | 3.125 | 6.25 | 3.125 | 6.25 |
| ZQ | 12.5 | 25 | 3.125 | 12.5 | 6.25 | 25 |
| YHJ | 12.5 | 50 | 6.25 | 25 | 12.5 | 25 |

MIC, Minimal inhibition concentrations. MBC, Minimal bactericidal concentration.

**Table S4.** Molecular docking binding energies of selected constituents with target enzymes.

| Binding energy (kcal/mol) | *E. coli* | | *S. aureus* | |
| --- | --- | --- | --- | --- |
|  | 1KZN | 2OMF | 2XCT | 1JIJ |
| D-Limonene | −5.8 | −5.2 | −5.0 | −5.7 |
| β-Phellandrene | −5.7 | −5.0 | −5.0 | −5.5 |
| Eucalyptol | −4.6 | −4.7 | −4.8 | −4.7 |
| Linalool | −4.8 | −4.2 | −4.3 | −4.9 |


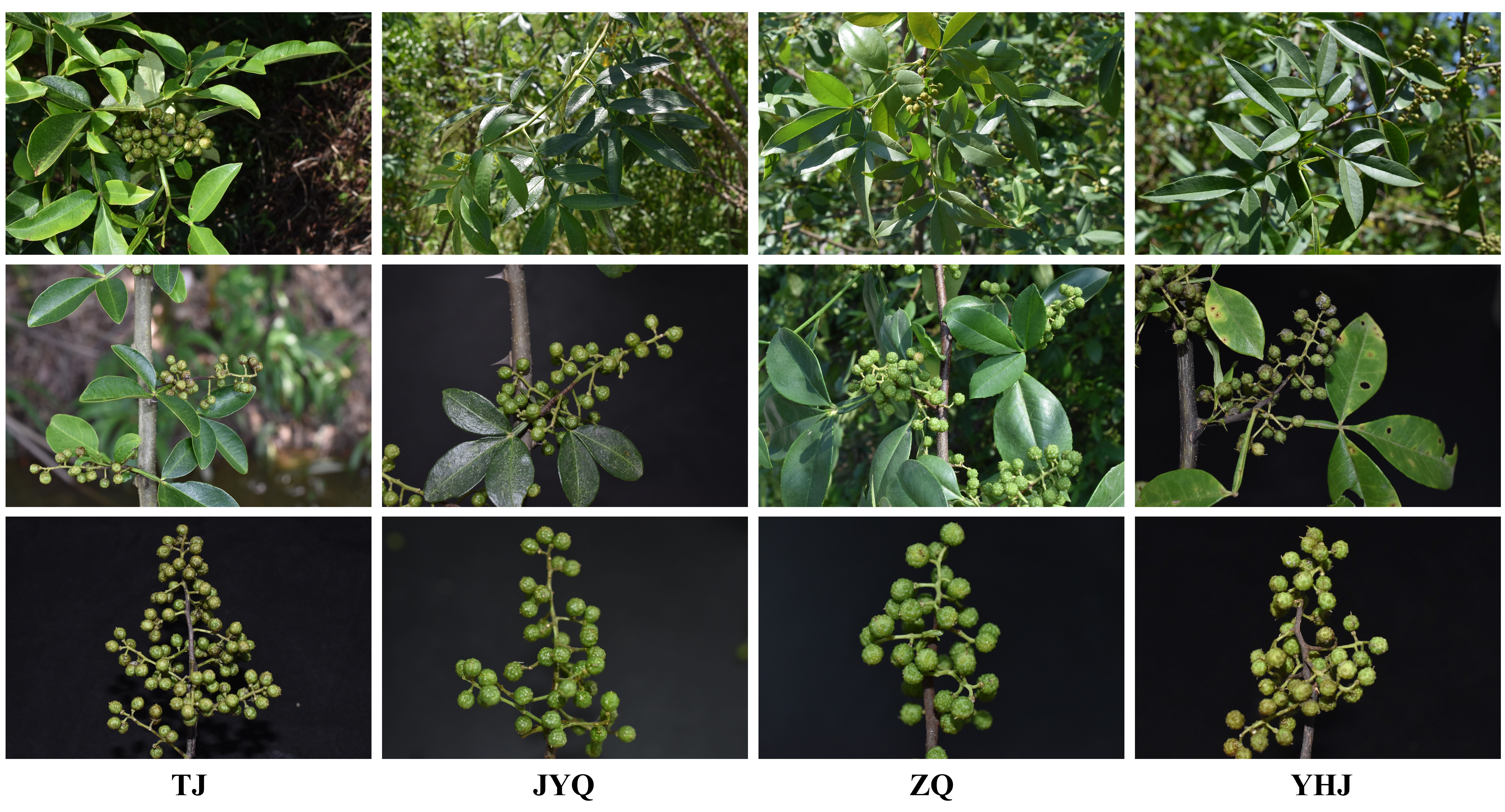


**Fig.** **S1.** Morphological characteristics of leaves, fruiting branches, and fruit clusters of four cultivars of *Zanthoxylum armatum*.


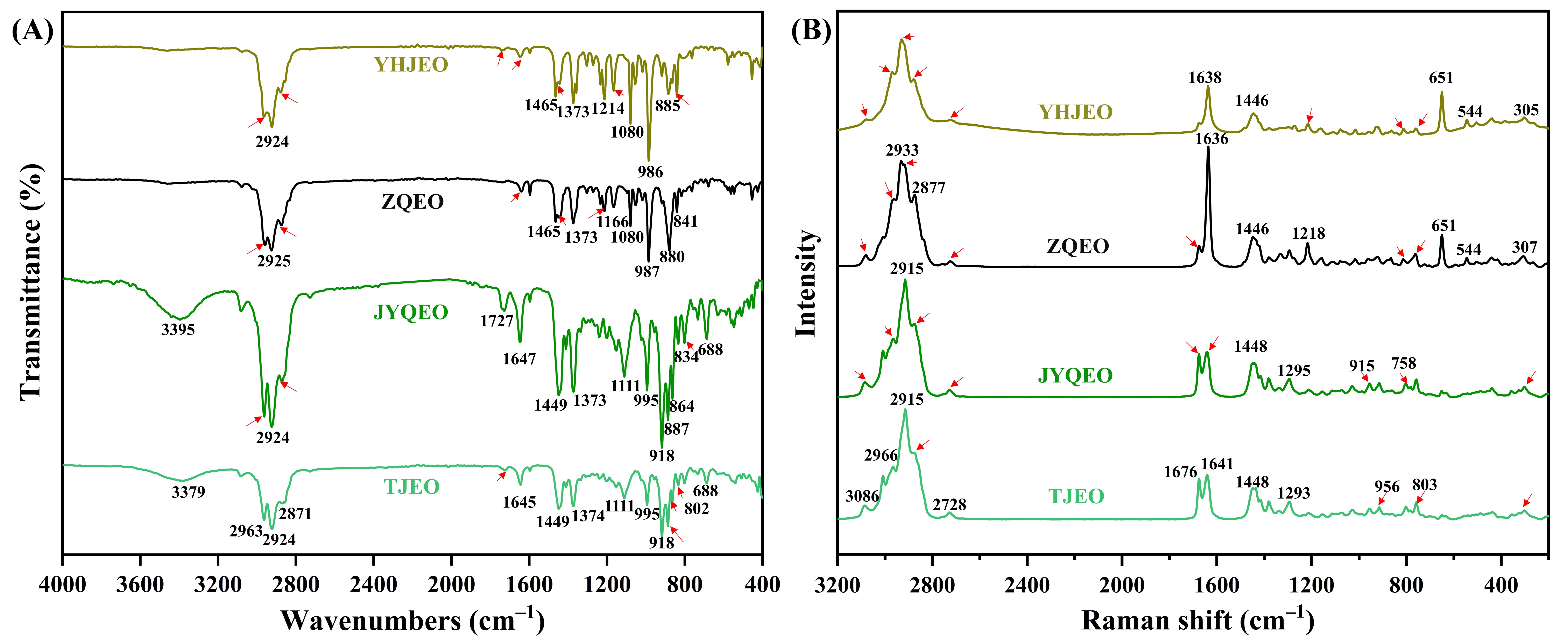


**Fig.** **S2.** FT-IR spectra (A) and Raman spectra (B) of four ZAEOs.


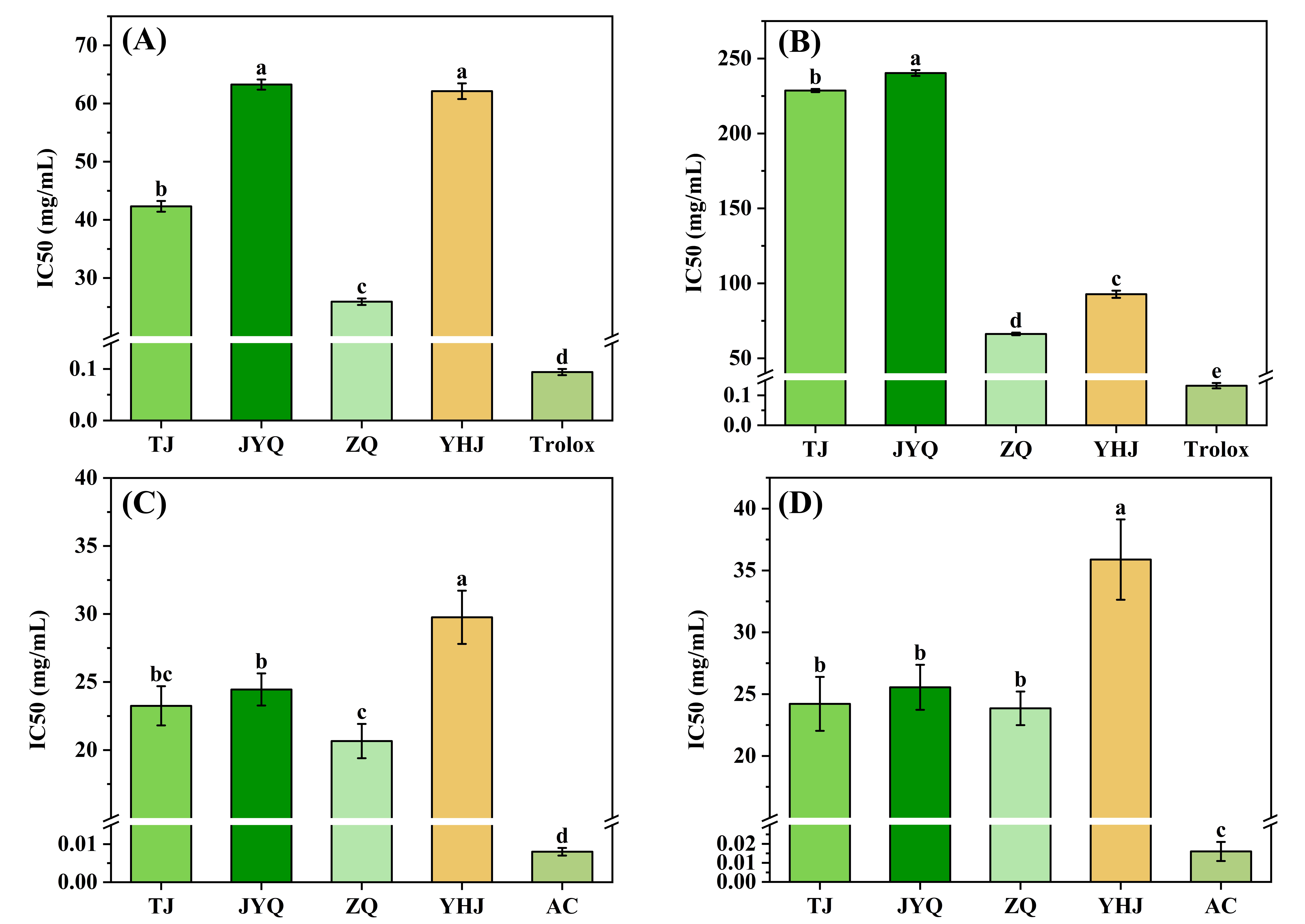


**Fig. S3.** Comparison of antioxidant and antidiabetic activities among four ZAEOs. IC50 values for ABTS radical scavenging activity (A). IC50 values for DPPH radical scavenging activity (B). IC50 values for α-Glucosidase inhibitory activity (C). IC50 values for α-Amylase inhibitory activity (D). IC50: Half maximal inhibitory concentration. Lowercase letters indicate significant differences (*p* < 0.05) among ZAEOs. Error bars represent standard deviation (*n* = 3).


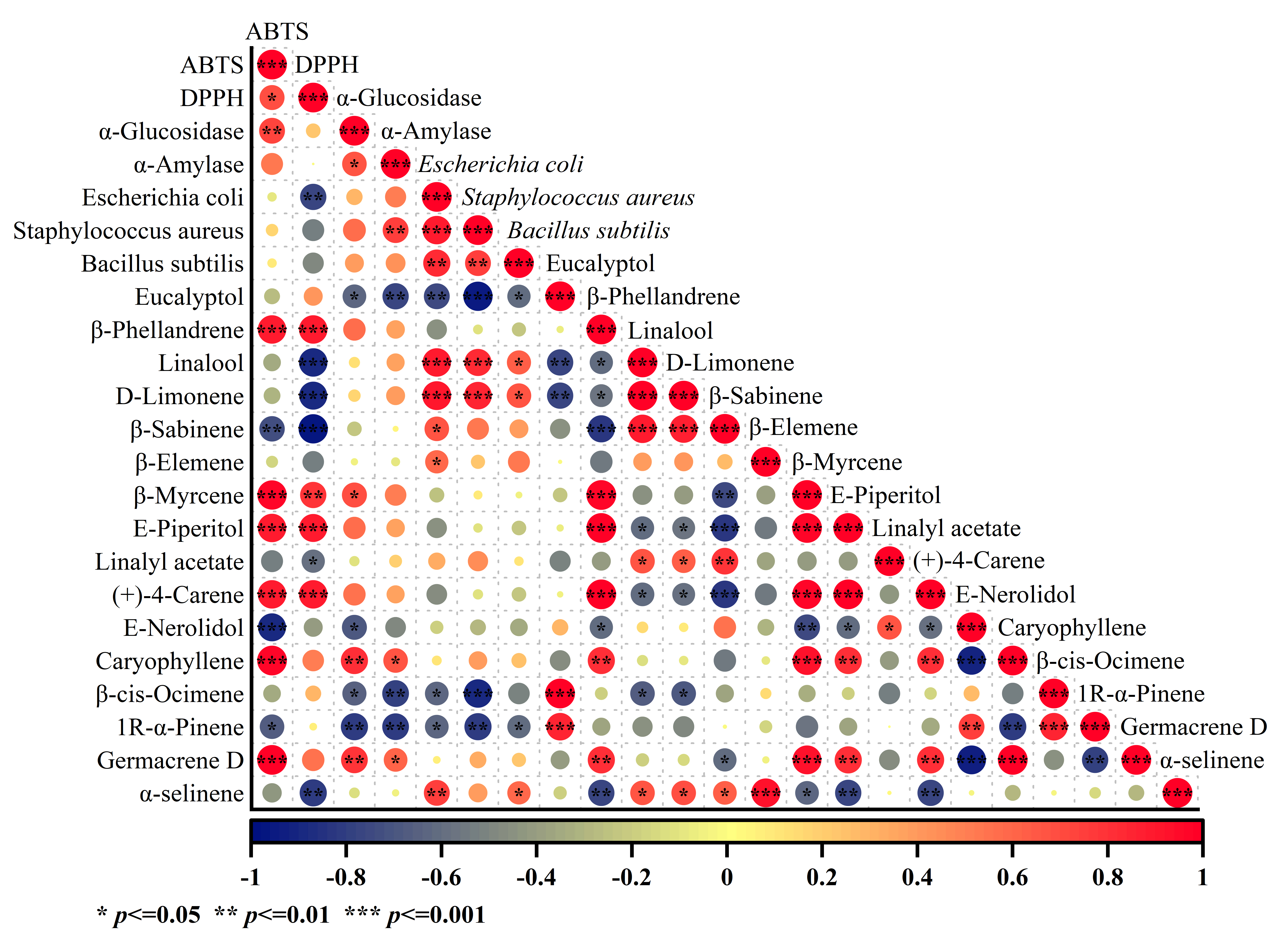


**Fig. S4.** Hierarchically clustered heatmap derived from the correlation matrix of studied variables in ZAEOs.


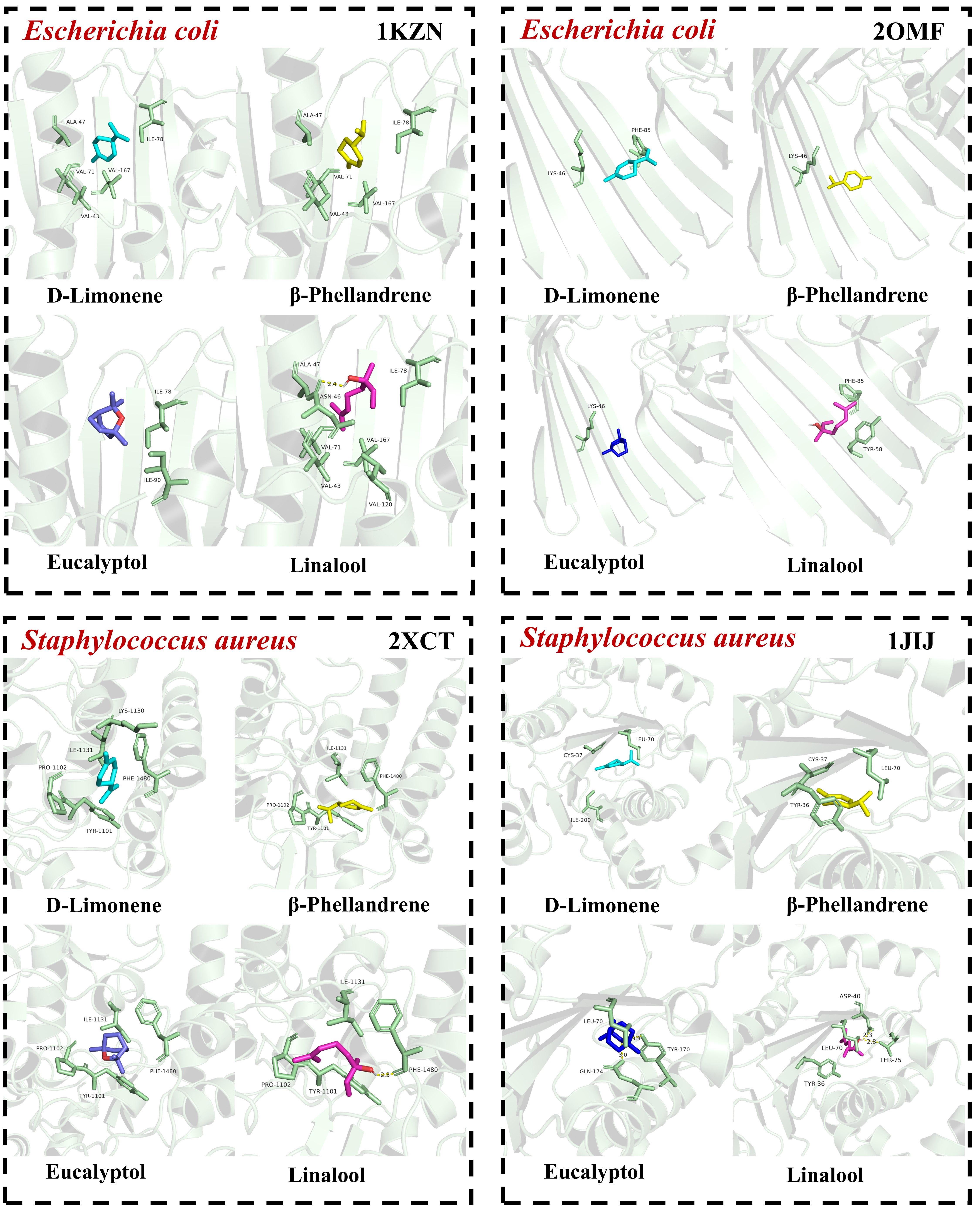


**Fig. S5.** Detailed 3D conformations of interactions between active sites of four proteins and D-limonene, β-phellandrene, eucalyptol, and linalool. In the detailed 3D conformations, the protein is depicted as a light green cartoon model. Ligands are displayed as stick models: D-limonene (cyan), β-phellandrene (yellow), eucalyptol (blue), and linalool (magenta). Residues interacting with ligands are highlighted as green sticks.
